# *In Vivo* Magnetic Enrichment, Photoacoustic Diagnosis, and Photothermal Purging of Infected Blood Using Multifunctional Gold and Magnetic Nanoparticles

**DOI:** 10.1371/journal.pone.0045557

**Published:** 2012-09-26

**Authors:** Ekaterina I. Galanzha, Evgeny Shashkov, Mustafa Sarimollaoglu, Karen E. Beenken, Alexei G. Basnakian, Mark E. Shirtliff, Jin-Woo Kim, Mark S. Smeltzer, Vladimir P. Zharov

**Affiliations:** 1 Phillips Classic Laser and Nanomedicine Laboratories, University of Arkansas for Medical Sciences, Little Rock, Arkansas, United States of America; 2 Arkansas Nanomedicine Center, University of Arkansas for Medical Sciences, Little Rock, Arkansas, United States of America; 3 Department of Microbiology and Immunology, University of Arkansas for Medical Sciences, Little Rock, Arkansas, United States of America; 4 Department of Pharmacology and Toxicology, University of Arkansas for Medical Sciences, Little Rock, Arkansas, United States of America; 5 Department of Molecular Pathogenesis, School of Dentistry, University of Maryland-Baltimore, Maryland, United States of America; 6 Bio/Nano Technology Laboratory, Institute for Nanoscience and Engineering, University of Arkansas, Fayetteville, Arkansas, United States of America; 7 Department of Biological and Agricultural Engineering, University of Arkansas, Fayetteville, Arkansas, United States of America; The Scripps Research Institute and Sorrento Therapeutics, Inc., United States of America

## Abstract

Bacterial infections are a primary cause of morbidity and mortality worldwide. Bacteremia is a particular concern owing to the possibility of septic shock and the development of metastatic infections. Treatment of bacteremia is increasingly compromised by the emergence of antibiotic resistant strains, creating an urgent need for alternative therapy. Here, we introduce a method for *in vivo* photoacoustic (PA) detection and photothermal (PT) eradication of *Staphylococcus aureus* in tissue and blood. We show that this method could be applicable for label-free diagnosis and treatment of in the bloodstream using intrinsic near-infrared absorption of endogenous carotenoids with nonlinear PA and PT contrast enhancement. To improve sensitivity and specificity for detection of circulating bacteria cells (CBCs), two-color gold and multilayer magnetic nanoparticles with giant amplifications of PA and PT contrasts were functionalized with an antibody cocktail for molecular targeting of *S. aureus* surface-associated markers such as protein A and lipoprotein. With a murine model, the utility of this approach was demonstrated for ultrasensitive detection of CBCs with threshold sensitivity as low as 0.5 CBCs/mL, *in vivo* magnetic enrichment of CBCs, PT eradication of CBCs, and real-time monitoring of therapeutic efficacy by CBC counting. Our PA-PT nano-theranostic platform, which integrates *in vivo* multiplex targeting, magnetic enrichment, signal amplification, multicolor recognition, and feedback control, could be used as a biological tool to gain insights on dissemination pathways of CBCs, infection progression by bacteria re-seeding, and sepsis development and treatment, and could potentially be feasible in humans, especially using bypass schematic.

## Introduction

The risk posed by the growing prevalence of antibiotic resistance is a pressing threat to public health, making bacterial infections a major problem worldwide [1]. Bacteria penetrate the skin (e.g., wound, catheters, or transfusion device) and are introduced into the normally sterile bloodstream leading to bacteremia. Bacteremia is one of the most devastating types of infections [2], and is commonly associated with *Staphylococcus aureus*, *Escherichia coli*, *Streptococcus*, *Pseudomonas*, and *Haemophilus*. The worldwide mortality rate from sepsis becomes comparable with the death from cardiovascular diseases. Hospital-acquired microbial infections are the fourth leading cause of death in the US, a problem exacerbated by the rapid rise in antibiotic resistant strains. The pathogen most likely to be associated with a fatal outcome is *S. aureus* [3]–[4]. Particularly problematic are methicillin-resistant *S. aureus* (MRSA) strains, which have a mortality rate in bacteremic patients as high as 30% [5]. Blood disseminates bacteria further to distant sites such as organs, joints, bone, heart valves, and inwelling medical devices, leading to the development of deep-seated, metastatic foci of infection. Bacteria from these metastatic foci can in turn reseed the bloodstream, making effective treatment of bacteremia even more difficult, particularly given the increased likelihood of the development of antibiotic resistance with prolonged antibiotic therapy.

One approach to addressing this problem is the development of new antimicrobial agents, but this is a time-consuming, costly process that has been de-prioritized by pharmaceutical companies and is currently at a historic low [6]. An attractive alternative would be to develop therapeutic strategies for the physical destruction of bacterial pathogens irrespective of their antibiotic resistance status. Possibilities in this regard include photodynamic therapy (PDT) using light in the presence of a photosensitizer and oxygen to produce a highly reactive singlet of oxygen that yields bacterial damage [7]–[14]. The efficacy of PDT, however, is dependent on tissue oxidation and bacterial infections in hypoxic environments, as seen in the late stages of osteomyelitis, may not be treatable with PDT [15]. Sonodynamic therapy [16]–[22] using ultrasound-triggered cavitation alone (see review in [21]), or in combination with PDT could be promising approach, especially by providing synergy of sono and PDT treatment [20]–[22]. As a possible alternative, nanotechnology using nanoparticles (NPs) of different composition, shapes or sizes (e.g., gold nanoshells, nanorods, magnetic NPs [MNPs], carbon nanotubes, or golden carbon nanotubes [GNTs]) have been extensively explored either as imaging contrast agents, as a transformer of various energy (e.g., laser, ultrasound, and radio-waves) to thermal and accompanied therapeutic effects (e.g., nanobubbles) or as the vehicles for drug delivery [23]–[35] using continuous wave (CW) [24] and pulse [23] lasers. Initially these methods have been investigated primarily in the context of cancer [23]–[29]. In particular, we introduced pulsed photothermal (PT) therapy of individual tumor cells targeted by gold NPs [23] and extended this approach to *in vivo* detection and killing of circulating tumor cells (CTCs) [25] with potential for multiplex CTC targeting using ultrasharp rainbow nanoparticles [27]. Using nanoparticles, we have also confirmed the efficacy of PA and PT methods for the in vitro detection and killing of single bacterial cells including *S. aureus* [30] and *E. coli* [31]. Later, these and similar approaches were explored for other microorganisms *in vitro* ([32]–[35], see review in [33]); however these methods have not been tested for bacteremia *in vivo*. We believe that mechanistic, if not biological, analogies exist in the progression of infection and cancer metastasis by circulating bacterial cells (CBCs) and CTCs, respectively. While the role of CTCs has already been investigated for metastasis prognosis and therapy [36], little progress has been made in CBC-related diseases. Assuming that CTCs in cancer and CBCs in bacteremia share the properties of being blood-borne agents that require selective destruction, we applied the *in vivo* PA-PT nano-theranostics platform previously developed for CTCs [25] to CBCs. We demonstrate, using antibody-conjugated two-color magnetic and gold NPs, that the *in vivo* integration of advanced PA and PT techniques can be harnessed to achieve the non-invasive, targeted, highly sensitive diagnosis and effective therapy of circulating *S. aureus* irrespective of their metabolic or antibiotic resistance status.

## Results

### PA-PT-nano-theranostic molecular platform for targeting of bacteria in blood and tissue

**Figure 1:**
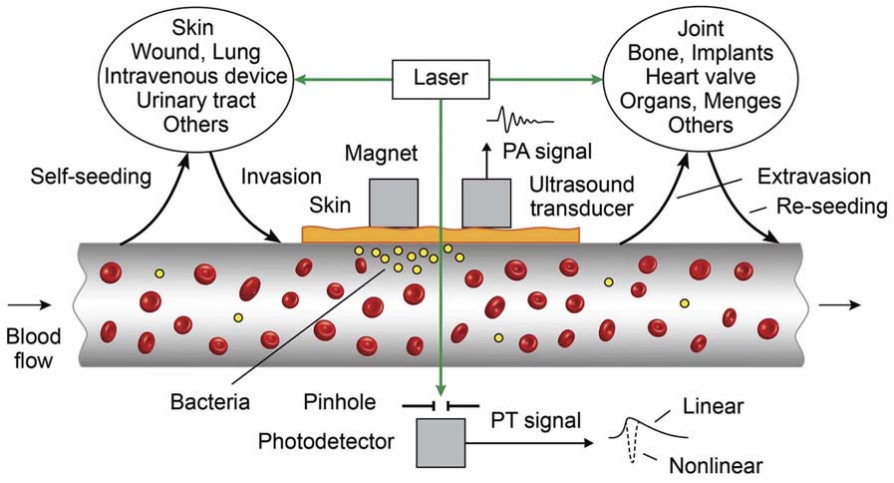
Principle of *in vivo* integrated PA-PT nano-theranostics of bacteria in blood and distant infected sites.

Based on our previous experience in developing a platform for integrated diagnosis and therapy (termed as theranostics) of CTCs [25], we modified this platform for theranostics of bacteria at a single cell level (Fig. 1). According to our proposed model, invasion of bacteria into the bloodstream creates CBCs that migrate from blood vessels and seed metastases in distant sites (i.e., new seeding). There is also a possibility that bacteria can reenter the circulation from these distant sites and disseminate to other sites (i.e. re-seeding). Thus, the repeated forward and reverse migrations between different sites by CBCs can disseminate the infection to different organs and blood, including the original site (i.e., self-seeding). If this hypothesis, which we call “self-seeding and reseeding by CBCs” (in analogy in botany and cancer [37], [38]), is correct, it could be the basis for a new form of bacterial diagnosis and therapy. Because CBC count is determined by a dynamic balance between bacterial invasion and extravasation in blood system, theranostics have to simultaneously target CBCs in the bloodstream and bacteria in primary and metastatic sites to exclude further CBC deposition. It is clear that CBC count can be used as a marker of therapy efficiency. In analogy to tumor-initiating (or stem cancer) cells [39]–[40], we cannot also exclude the presence of aggressive populations of CBCs, which may significantly contribute to bacterial colonization and drug resistance. Theoretically the targeted CBC could also be used as a “Trojan horse” to deliver therapeutics to metastatic and primary sites.

Based on our proposed model, we developed a new universal PA-PT-nano-theranostic platform for the single-bacterium-level targeted therapy of infections at various locations in typical dissemination pathways, (i.e. primary infected sites→invasion into blood→hematogenous spreading→extravasations→metastasis) with primary focus on CBCs directly in the bloodstream during bacteremia. To detect CBCs, the selected blood vessels are irradiated with a short laser pulse followed by time–resolved detection of laser-induced acoustic waves (referred to as PA signals) from CBCs with an ultrasound transducer attached to skin near the vessels (Fig. 1). The physical mechanism of this PA flow cytometry (PAFC) is associated with fast (picosecond scale) nonradiative relaxation of absorbed laser energy into heat and then the thermoelastic generation of acoustic waves [40]. Laser radiation is delivered to tissue by using a microscope schematic (Fig. S1) with a customized condenser to create the desired linear beam shapes. A fiber with a miniature tip and cylindrical optics is also available [40].

PAFC was integrated with a PT flow cytometry (PTFC) [34], [40] and PT therapy of CBCs. In the PTFC thermal lens mode, a laser–induced refractive heterogeneity in CBCs causes defocusing of a collinear laser probe and hence a reduction in the beam's intensity at its center, as detected by a photodiode with a pinhole referred to as PT signals. These signals in a linear mode (i.e., without notable cell photodamage) represent a standard positive peak associated with rapid (pico-nanosecond scale) cell heating and a slower, microsecond scale tail corresponding to CBC cooling as a whole (Fig. 1, bottom, right). Laser-induced nanobubbles around overheated absorbing zones manifested through appearance of nonlinear sharp PT negative peaks are used as PA signal amplifier and CBC killer [30]–[31]. This platform could enable us to explore both intrinsic CBC markers [41] and NPs as PA and PT molecular agents.

### Label-free detection and killing of *S. aureus in vitro* and *in vivo*

**Figure 2:**
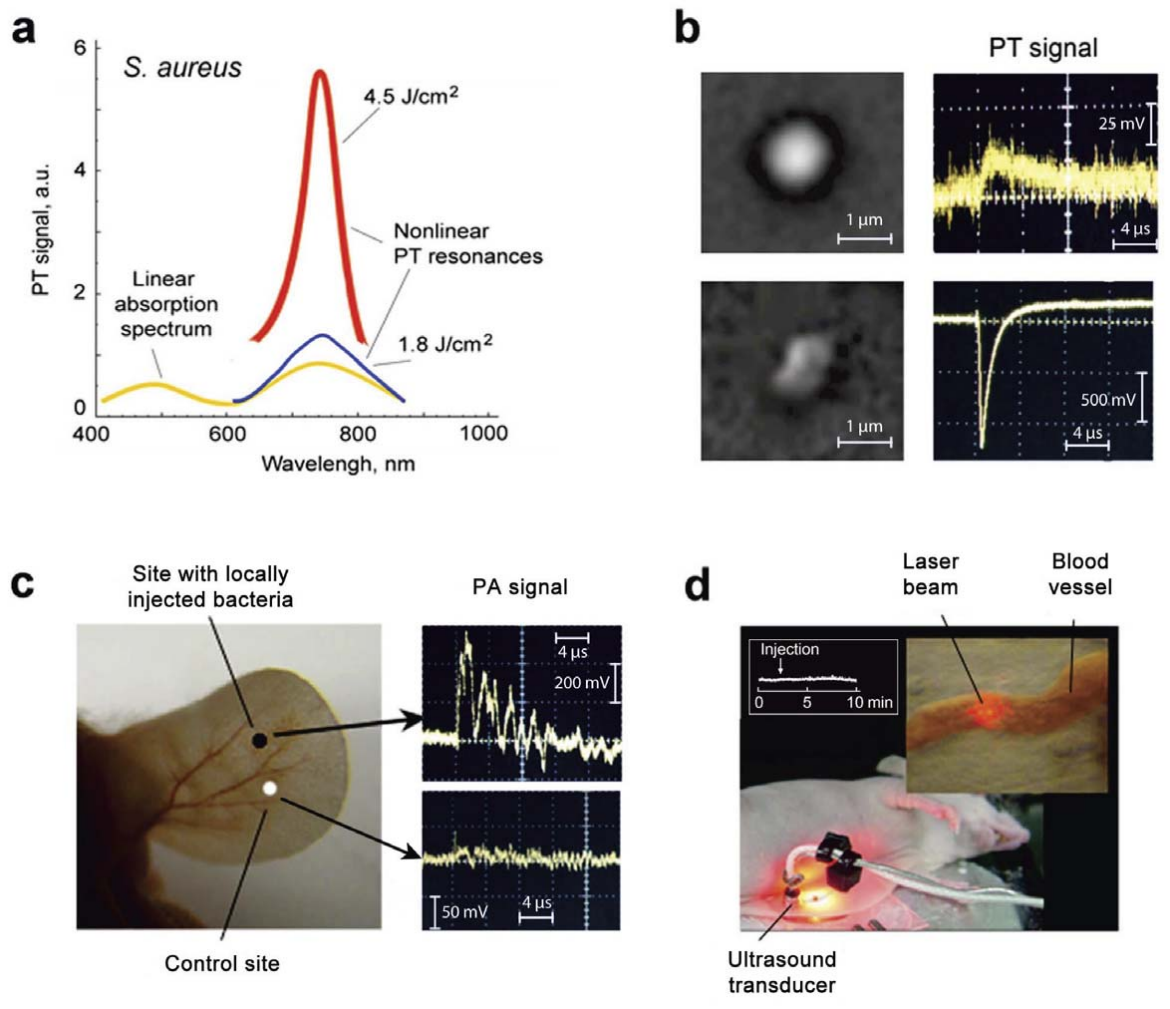
Label free PA diagnosis and PT therapy of *S. aureus*. (**a**) Linear absorption (yellow curve) and nonlinear PT spectra of bacteria in solution (10^6^ CFU/mL) in 1 cm optical pathway at two energy fluence levels: 1.8 J/cm^2^ (blue curve) and 4.8 J/cm^2^ (red curve). The average standard deviation in PT data for each wavelength at automated laser spectral scanning was 21%. (**b**) High-resolution optical image (top, left) and linear PT signal (top, right) from single intact *S. aureus* at laser wavelength of 740 nm and low energy fluence (0.9 J/cm^2^), and high-resolution optical image of photodamaged *S. aureus* (bottom, left) and nonlinear PT signal associated with laser-induced nanobubbles at high laser fluence of 10 J/cm^2^ (bottom, right). The intact bacteria had round shapes compared to irregular shapes of the photodamaged bacterium. (**c**) *In vivo* label-free PA detection of bacteria in mouse ear (left) showing increase of PA signal (right, top) compared to control measurement without bacteria (right, bottom). The infected site was modeled by injection of 20-µL bacteria suspension in concentration of 10^4^ CFC/mL in mouse ear. Laser parameter: wavelength, 741 nm; energy fluence, 1 J/cm^2^. (**d**) Laser irradiation of ∼300-µm abdominal blood vessel (callout) in skin-fold mouse model followed by detection of acoustic waves with the ultrasound transducer closely attached to laser beam.

To establish a baseline for our theranostic studies, we first assessed the PA and PT properties of intrinsic chromophores of *S. aureus*. The spectrophotometry of a bacterial suspension in phosphate-buffered saline revealed two absorption peaks near 462 nm and 741 nm (Fig. 2a, yellow curve), possibly associated with carotenoids [41]. PT spectroscopy at low laser energy confirmed this finding (Fig. 2a, blue curve). Laser-induced nanobubbles at high laser energy [30]–[31] led to a nonlinear ∼10-fold PA signal amplification accompanied by spectral narrowing of the absorption band (Fig. 2a, red curve). Spectral sharpening and amplification of both PA and PT responses improved spectral specificity and threshold sensitivity to one bacterium in the irradiated volume, as well as PT killing of an individual bacterium. Thus, even low laser energy was sufficient for the noninvasive detection of a single bacterium in the irradiated volume (Fig. 2b, top), while high-energy irradiation led to cell death as confirmed by conventional viability assay, specific changes in PT signal shape due to the appearance nanobubble-associated negative peaks, and cell architecture (Fig. 2b, bottom). Then we attempted to replicate this *in vivo* focusing on a localized infection in the murine ear. We found that the ratio of PA signals to background was in the range of 3–6 (Fig. 2c). However, in a bacteremia model, in which *S. aureus* was introduced directly into the bloodstream of a mouse, with irradiation being done through the abdominal wall and PA signals examined using a closely attached transducer (Fig. 2d), we determined that the label-free PA detection of blood-borne *S. aureus* cells was difficult due to the influence of the blood background.

### Nanoparticle characterization and cytotoxicity evaluation

**Figure 3:**
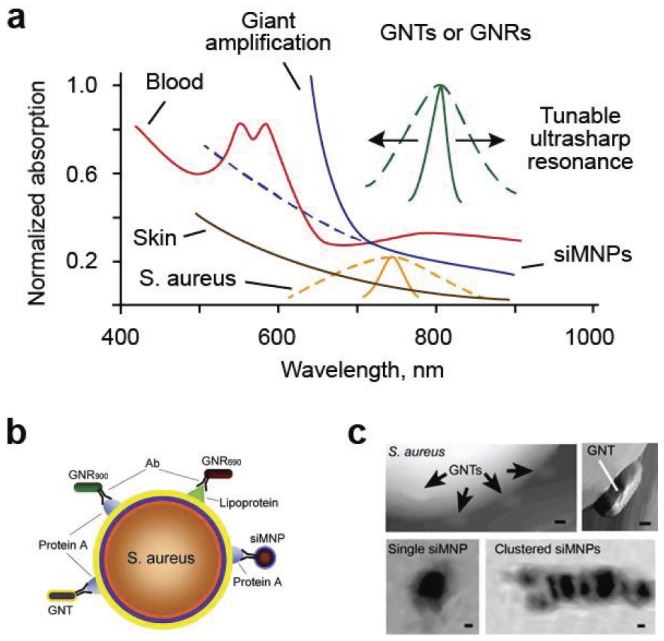
Schematic of label-free and targeted theranostics of *S. aureus*. (**a**) Absorption spectra of blood (red), skin (orange), *S. aureus* (yellow), and different NPs: GNTs, GNRs (green), and siMNPs (blue). Dashed and solid lines indicate averaged linear and nonlinear PA spectra, respectively. (**b**) Multiplex targeting *S. aureus* surface biomarker protein A (Spa) and lipoprotein (Lpp) by siMNPs, GNRs and GNTs functionalized with either anti-Spa or anti-Lpp Abs. (**c**) Atomic force microscopy (AFM) images of GNTs on the surface of an *S. aureus* cell (top) and transmission electronic microscopy (TEM) images of single and clustered siMNPs with a ∼30-nm magnetic core and ∼10-nm silica layer (bottom, left and right, respectively). Scale bars, 10 nm.

To enhance both PT and PA contrast in the near-infrared (NIR) range within tissue window transparency (650–1100 nm), we explored different NPs including magnetic NPs (MNPs), gold nanorods (GNRs), carbon nanotubes (CNTs), and golden carbon nanotubes (GNTs) (Fig. 3a, Figs. S2, S3, S4, S5, S6, S7, S8). We observed that many NPs had a tendency to aggregate (Fig.S5, S6, S7). If the degree of NP's aggregation could not be improved by sonication, filtration and other common procedures [28], [29], [34], the NPs were excluded from further studies. As a result we finally selected spectrally tunable GNTs [42] and GNRs as well as silica-coated magnetic MNPs (siMNPs) for multiplexing targeting (Fig. 3a–c, Figs. S2, S3). To increase specificity, these NPs were functionalized by conjugation to antibodies (Abs) specific for *S. aureus* protein A (Spa) and/or lipoprotein (Lpp) (Fig. 3b), both of which are highly expressed in *S. aureus* and absent in mammalian cells [43].

The low toxicity of MNPs and GNTs was already demonstrated in previous studies [25], [42]. In particular, cell viability and proliferation assays confirmed no apparent adverse toxic effects on living cells after their exposure to various concentrations of GNTs (0.05–0.5 mg/mL) for 10 days [42]. This indicates no or at least minimal GNT cytotoxicity at the typical doses that was used in this study (i.e. ≤1 µg/mL). By comparison, a CNT dose of 0.5 mg/mL produced notable toxicity in 6–12 hours. In addition, no marked changes in mouse behavior and blood vessel function (e.g. size, flow velocity, and hematocrit) were observed over a 1-month exposure to the GNTs (50 nM per day) [42].

**Figure 4:**
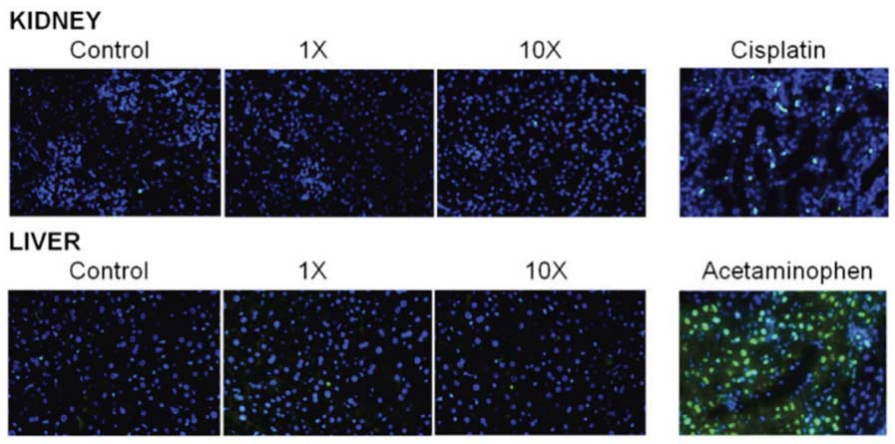
Assessment of systemic toxicity with Ab-conjugated GNRs in kidney and liver. The 1× and 10× designations refer to approximately 10^11^ and 10^12^ Ab-conjugated GNR NPs, respectively, injected into the bloodstream of mice via the tail vein. Samples from liver and kidney at 24 h after NP injections were assayed for evidence of apoptotic and/or necrotic damage using the TUNEL assay (green). Nuclei were counterstained with DAPI (blue). Cisplatin (20 mg/kg) was used as a positive control for kidney damage, while acetaminophen (300 mg/kg) was used as a positive control for liver damage.

To estimate possible toxicity of Ab-conjugated GNRs, we have determined their effects on two major excreting organs, liver and kidney, which are the common targets for nanoparticle toxicity [44], [45]. The Ab-conjugated GNRs were injected into the bloodstream of mice via the tail vein, with the 1× and 10× designations referring to approximately 10^11^ and 10^12^ NPs, respectively. These amounts were chosen based on the efficiency of Ab conjugation and in an attempt to maximize exposure without exceeding a 100-µL injection volume. After 24 h, mice were humanely sacrificed and samples from kidney and liver assayed for evidence of apoptotic and/or necrotic damage using the TUNEL assay (Fig. 4, green). Nuclei were counterstained with DAPI (Fig. 4, blue). Cisplatin (20 mg/kg) was used as a positive control for kidney damage, while acetaminophen (300 mg/kg) was used as a positive control for liver damage as described previously [46], [47].

### *In vitro* multiplex targeting of *S. aureus* cells using gold NPs conjugated with antibody cocktail

**Figure 5:**
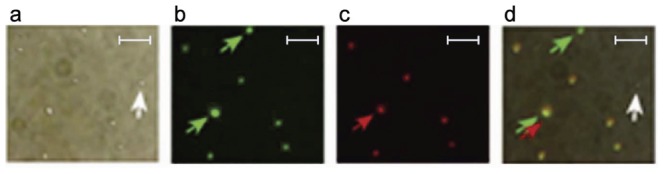
Molecular targeting of *S. aureus* with fluorescent dyes and siMNPs functionalized with anti-Spa and anti-Lpp Abs. (**a–c**) Optical (a) and fluorescence images of bacteria targeted with FITC-labeled anti-Spa Abs (green, b) or phycoerythrin (PE)-labeled anti-Lpp (SA0486) Abs (red, c). (**d**) Overlaid image that confirms co-localization of Abs. Green and red arrows indicate Spa-positive and Lpp-positive bacteria, respectively, and the white arrow indicates a rare unlabeled bacterium. Scale bars, 5 µm.

Comparison of optical and fluorescence images of bacteria (Fig. 5) labeled (30 min, 37°C) with NPs functionalized with Ab-fluorescent dye (FITC and PE) conjugates revealed that 82.5±2.49% of bacteria were detectable with the anti-Spa Abs (termed anti-Spa), 81.2±1.90% with the anti-Lpp Abs (termed anti-Lpp), and 89.7±2.90% with both Abs, the latter confirming the increased targeting efficiency obtained with multiple Abs. The NP properties were assessed by atomic force microscopy (AFM), transmission electron microscopy (TEM), optical microscopy (Fig. 3c, Figs. S2, S3, S10) and PA/PT microscopy/cytometry [42], the latter demonstrating a 30 to 50-fold signal increase by comparison to unlabeled bacterial cells.

The comparison of PA/PT signals from unlabeled control bacteria and bacteria labeled with anti-Spa conjugated to GNR^900^, anti-Lpp conjugated GNR^690^, or a combination of the two GNRs confirmed targeting efficiencies of 91.1±2.50%, 89.0±2.20%, and 96.4±3.20% respectively. Compared to fluorescent tags, the increased NP targeting efficiency can be explained by the higher PT/PA contrast, which requires an average of 1,000 GNRs per cell. Decreasing the number of Abs-conjugated GNRs per cell to 100 still provided detectable signals, at least *in vitro*, without a decrease in targeting efficiency. Even at 10 GNRs per cell, detectable signals were obtained, although targeting efficiency decreased to <60%. Similar efficiency was observed with Abs conjugated GNTs^900^ (92.4% with 100 GNT per cell). However, MNPs provided lower PT contrasts (e.g., 75–85% for 1000 MNPs/cell). Nonspecific binding for most NPs without Abs was in the range of 5–7%.

Mouse blood samples were spiked with *S. aureus* and treated with NP-Abs-fluorescent dye conjugates either alone or in a cocktail (GNR^690^/GNR^900^ proportion of 50∶50) under static conditions. Targeting efficiency was estimated by fluorescent imaging and PT techniques using optical imaging for identification of small *S. aureus* among red blood cells. Labeling of bacteria in mouse blood decreased targeting efficiency by 10–14% on average and increased nonspecific binding to 8–10% (Table 1).

**Table 1:** Labeling efficiency (%) of *S. aureus* (1 h, 37°C) in static condition *in vitro* obtained with fluorescent and PA (in brackets) techniques.

Samples	Antibodies (Nanoparticles)		
	Anti-LPP(GNR^900^)	Anti-Spa(GNR^690^)	Anti-LPP(GNR^900^)+Anti-Spa(GNR^690^)
Bacteria in PBS	81.2±1.9% (91.1±3.1%)	82.5±2.4% (89.0±2.8%)	89.7±2.9% (96.4±3.2%)
Bacteria in mouse blood	67.7±1.8%	72.1±2.0%	79±2.6%
Mouse blood with no bacteria (control)	8±1.3%	6±1.2%	10±1.6%

### Giant nonlinear signal amplification in single siMNPs and magnet-induced siMNP clusters

**Figure 6:**
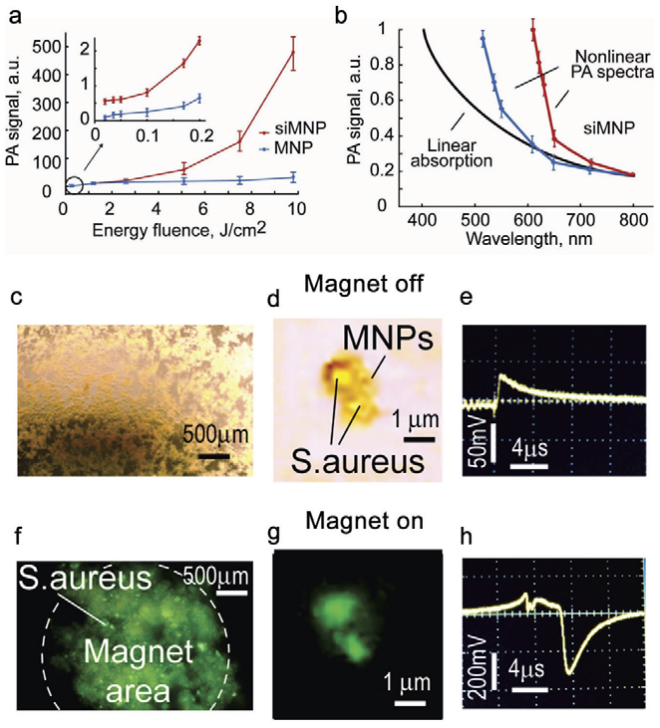
Giant nonlinear signal amplification in single siMNPs and magnet-induced siMNP clusters. (**a**) Nonlinear PA signal amplification from siMNPs (red curve) compared to MNPs without a silica layer (blue curve) at 639 nm. (**b**) Nonlinear PA spectra of siMNPs (blue and red curves) at 0.3 and 5 J/cm^2^ energy fluences. Black curve indicates the linear absorption spectrum. PA spectra were normalized on absorption spectra at 800 nm. The error bars represent the standard deviations in five measurements. (**c–h**) Optical (c, d) and fluorescent (f, d) images of many (c, f) and two (d, g) *S. aureus* labeled by 30-nm siMNPs with FITC, and PT signals from two labeled bacteria before (e) and after (h) magnet exposure for 3 minutes. Laser parameters: wavelength, 640 nm; energy fluence, 200 mJ/cm^2^; pulse rate, 10 Hz.

The use of MNPs offers the advantage of magnetic capture of cells [25]. However, the NIR contrast with MNPs was lower (10–30-fold) by comparison to GNTs and GNRs (Fig. 3a). Taking into account the previously discovered linear PA signal enhancement using gold nanoparticles with a dense layer of polymer [48] and silica [49], we evaluated nonlinear PA/PT properties of MNPs with silica layer (siMNPs). At low laser energy (*i.e.*, in linear mode), siMNPs demonstrated 1.8 to 2.5-fold increased PA signals compared to MNPs without silica layer. At higher laser energy, we observed a 20 to 35-fold nonlinear PA/PT signal amplification (Fig. 6a), a phenomenon referred to as giant amplification. This can be explained by the favorable thermal acoustic and bubble-related properties of siMNPs for the generation of nonlinear PT and PA effects including silica-induced rigid restriction for thermal expansion of the heated magnetic core (PA spherical piston model) [48]. We also observed the amplified PA spectra within the NIR window tissue transparency due to more profound bubble-associated nonlinear effects at higher MNP absorption (Fig. 6b). In addition, the magnet-induced clustering of siMNPs provided a nonlinear PT and PA signal increase due to the enhanced nanobubble formation around strongly-absorbing siMNP clusters. Specifically, the attachment of a magnet tip to the bottom of microscopic slide with MNPs with FITC (Fig. 6c) led to accumulation of MNPs to the area of the magnet. This was visualized even by the naked eye as a dark spot and verified by fluorescent microscopy (Fig. 6f), which showed increases in fluorescent intensity from magnet-induced MNP clusters. Similar effects were obtained from two bacteria labeled by MNP-FITC conjugate (Figs. 6d,g). The PT signals from the area with high local concentration of MNPs after magnetic action (Fig. 6h) was significantly higher (10–20 fold) compared to the PT signals of the intact sample with changed temporal shape (Fig. 6e). This suggests that magnetic-induced clustering of MNPs within individual bacteria yielded enhancement of PT signals accompanied by nanobubble formation around the overheated local zone with increased absorption. The appearance of the local zones with dense MNPs (i.e., clusters) is likely due to the accumulation of MNPs under magnet on bacterial surface. These data are in agreements with results obtained for cancer stem cells labeled with MNPs [50].

### *In vivo* noninvasive PA detection of CBCs targeted by gold NPs

**Figure 7:**
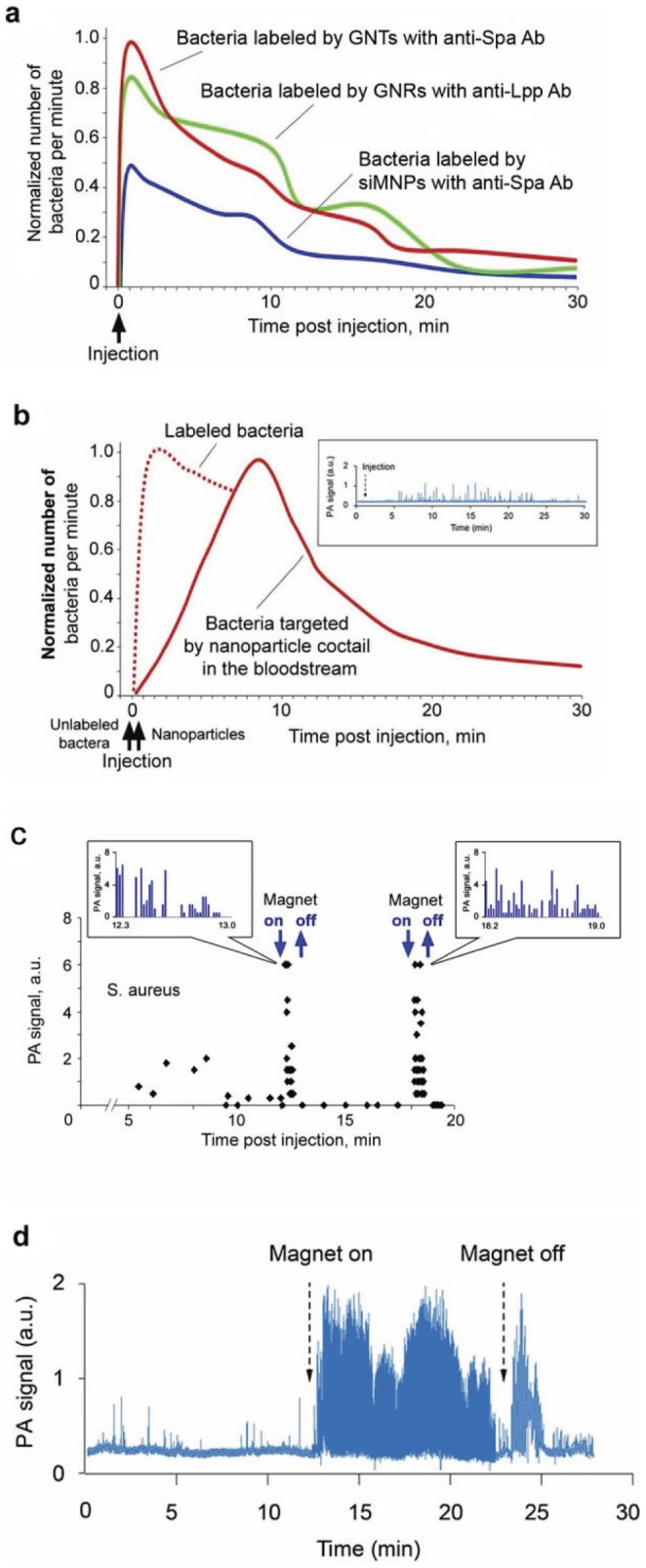
*In vivo* detection of CBCs with Ab-conjugated NPs and magnet-induced CBC capturing and signal amplification. (**a**) *In vivo* PA monitoring of CBCs labeled with Ab-functionalized NPs *in vitro* prior to injection. (**b**) PA monitoring of CBCs labeled *in vivo* with a cocktail of functionalized NPs consisting of GNRs^690^ with anti-Lpp Ab and GNRs^900^ with anti-Spa Ab in a 50%∶50% proportion in 20 µL of phosphate-buffered saline (n = 3). The inset shows typical PA signal traces. Dashed line indicates PA monitoring observed when the same cocktail was used to label *S. aureus* cells *in vitro* prior to injection (similar to Fig. 7a). The average standard deviation for each time point in (a) and (b) is 18% (**c**) Verification of magnetic amplification of PA signals from bacteria labeled with 30-nm MNPs with anti-Spa Ab in rat mesentery. The inset shows typical PA signal traces. (**d**) *In vivo* real-time monitoring of PA signal dynamic from bacteria labeled *in vivo* by siMNPs with anti-Spa Ab before, during, and after magnetic field action in mouse ear. Laser parameters: wavelengths, 671 nm and 820 nm (a, b), 639 nm (c), 671 nm (d); energy fluence, 0.1 J/cm^2^.

To assess the utility of these methods *in vivo*, *S. aureus* cells were first pre-labeled with anti-Spa conjugated GNTs and/or anti-Lpp conjugated GNRs. Labeled bacterial cells (10^5^) were introduced by tail vein injection into mice, with PA signals monitored via a 50-µm blood vessel in the ear. Signals were detected within the first minute, reached a maximum within 3 minutes, and gradually declined over the next 20 minutes (Fig. 7a). When 10^5^ unlabeled *S. aureus* cells were injected followed by injection of the Ab-conjugated NP cocktail (10^9^), no PA signals were detected immediately after injection, but an increase in both PA signal number and amplitude was observed during the 10–15 minute period after injection of NPs (Fig. 7b). After reaching its maximum, there was a gradual decrease in signal intensity, with a clearance time similar to that observed with *in vitro* labeled bacteria (Fig. 7a). No PA signals were detected from NPs injected alone at concentration ≤10^9^. These data suggest successful *in vivo* targeting of *S. aureus* cells circulating in the bloodstream approximately 10 min after Ab-conjugated NP injection without any signal interferences from unbound NPs, which were below background noise.

### *In vivo* magnetic capturing and signal amplification from CBCs labeled with siMNPs

When bacteria were pre-labeled *in vitro* with 50-nm siMNPs (1 h, 37°C) and introduced by tail vein injection in rat, only rare PA signals were observed. However, when a magnet was applied initially to the mesentery vein as an ideal model for proof-of-principle, we immediately observed a significant increase in both the amplitude and rate of PA signals (Fig. 7c). Removal of the magnet led to a short-term increase in signal rate, likely due to the release of trapped bacteria, followed by the disappearance of strong signals. Subsequent attachment and removal of the magnet again led to similar phenomena. After verification using this invasive model with bacteria labeled *in vitro*, we repeated the experiments *in vivo* using 30-nm anti-Spa conjugated siMNPs and non-invasive application of the magnet to the skin overlying an ear vein. These studies were done sequentially, with unlabeled bacteria being introduced into the bloodstream via tail vein injection followed by the introduction of Ab-conjugated siMNPs. Magnet attachment led to a 10 to 15-fold amplification of PA signals (Fig. 7d). Slight fluctuations in PA signal amplitude and rate suggests a dynamic balance of bacteria near the magnet, with some proportion of trapped bacteria being randomly removed by shear forces. In general, the combination of PA signal amplification with Ab conjugated siMNPs and magnetic trapping of siMNP-labeled bacteria allowed more than a 100-fold enhancement of sensitivity, providing signals comparable to those observed with Ab-conjugated GNTs and GNRs.

The data with siMNPs is in agreement with our results previously obtained for CTCs labeled by MNPs [25]. Unbound siMNPs could be captured only in static condition. Introduction of free siMNPs in high concentration (10^11 si^MNPs/mL) and siMNP-labeled cells demonstrated that both siMNPs and siMNP-labeled cells were captured at flow velocity of ≤0.1 cm/sec, while only labeled cells were captures at higher velocity. Because magnetic force is proportional to particle number, the randomly distributed free siMNPs were more effectively removed by flow drag forces than the labeled cells that contained a much higher local MNP concentration (e.g., up to 100–500 MNPs in volume of one cell, ∼1 µm^3^ cell). Nevertheless, we observed that occasionally small siMNP aggregates can produce readable PA signals immediately after siMNP injection and can be captured if the magnet is on. Fortunately, siMNP aggregates were cleared usually within a few minutes compared to longer circulation of individual siMNPs. We applied the magnet 10–20 minutes after siMNP injection not only to avoid false positive signals by allowing siMNP aggregates to be cleared but also to provide enough time for bacteria to be labeled by siMNPs.

### Monitoring of bacteria extravasations and dissemination

**Figure 8:**
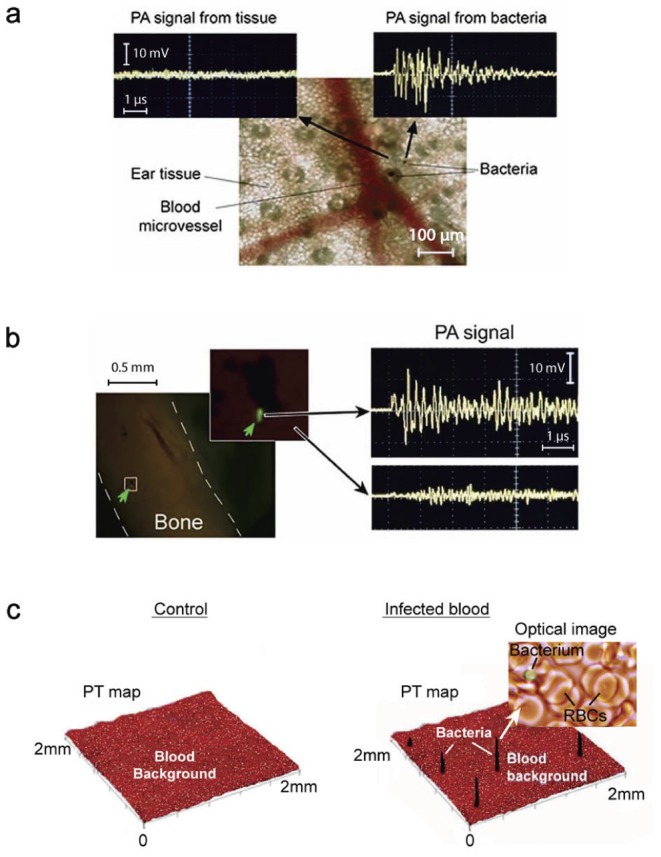
*In vivo* monitoring of labeled bacteria in tissue. (**a**) *In vivo* monitoring of bacterial extravasation from a mouse ear vein. (**b**) Arrest of circulating bacteria in mouse tibial bone labeled *in vitro* with 30-nm MNPs functionalized with FITC-conjugated Abs (left). The presence of bacteria in bone was confirmed by the appearance of PA signals after fluorescence-guided laser irradiation of green spots (right, top) compared to background signals from bone alone (right, bottom). (**c**) PT map of a 120-µm slide containing blood spiked with bacteria labeled with anti-Spa-Ab-conjugated GNTs^900^. Black peaks are associated with single bacteria as verified by optical imaging (callout). Laser parameters: wavelengths, 639 nm (a, b, c); energy fluence, 0.1 J/cm^2^.

Scanning of microvessels using a focused laser beam with a diameter of 10 µm (*in vivo* PA scanning cytometry) revealed rare (1–3 per one mm) stationary PA signals preferentially in small venules. This suggests the presence of non-circulating *S. aureus* cells attached to the vessel wall. In mouse ear vessels, the maximum signal number was reached 5–8 minutes after administration of bacteria and lasted for several hours with only a slight decrease in signal number. While this decrease could reflect the release of bacteria and their re-introduction back into the bloodstream, we also observed PA signals in the immediate vicinity (100–500 µm) of the vessels (Fig. 8a). This suggests extravasation of bacteria into adjacent tissues. This finding was confirmed by the examination of tissues one hour after injection of bacteria labeled with FITC and 30-nm anti-Spa conjugated siMNPs. In particular, fluorescent imaging of tibial bone *ex vivo* (Fig. 8b), as well as liver and spleen *in vivo* using microsurgery incision to provide access of optical fiber to these organs (data not shown), revealed strong PA signals generated from fluorescent spots, thus confirming the presence of *S. aureus* cells in these tissues.

### The sensitivity threshold

To estimate PA threshold sensitivity, mouse blood was spiked *ex vivo* with unlabeled and labeled bacteria at different concentrations (∼5, 30, and 100 cells per volume analyzed) and subjected to PA imaging on microscope slides. Even at maximum absorption near 740 nm, unlabeled bacteria did not provide detectable signals in these blood samples. Strong signals were observed only with NP-labeled bacteria as confirmed by optical imaging (Fig. 8c, Figs. S8, S9 and S10). By changing the laser beam size and blood sample thickness, the detection limit at a signal-to-noise ratio of 2 was estimated to be one bacterial cell with a beam diameter of 50 µm and thickness of 0.25 mm for anti-Spa-conjugated siMNPs and thickness of 0.4 mm for the Ab-conjugated GNRs.

The threshold sensitivity *in vivo* was estimated in the rat mesentery models using microscopic schematic. Bacteria were first labeled *in vitro* with GNRs, then injected intravenously in the rat blood circulation followed by PA monitoring of mesentery microvessels. Mesentery with clearly distinguishable blood vasculature in thin ∼10 µm mesentery tissue is an almost ideal model to assess individual vessels. Most bacteria were cleared during 10–20 minutes. However, later rare (a few signals per one hour) PA signals were still detected up to several hours later. After 5 hours when we observed next PA signal, blood was immediately drawn and was subject of PA examination in microscopic slides *ex vivo* (Fig. S10). Comparison of PA signal rate *in vivo* and *in vitro* demonstrated a sensitivity threshold of 0.5 CFU/mL, or approximately 10–15 bacteria in a rat's total blood volume (25 mL).

### *In vivo* real-time PA monitoring of targeted PT therapy of CBCs

**Figure 9:**
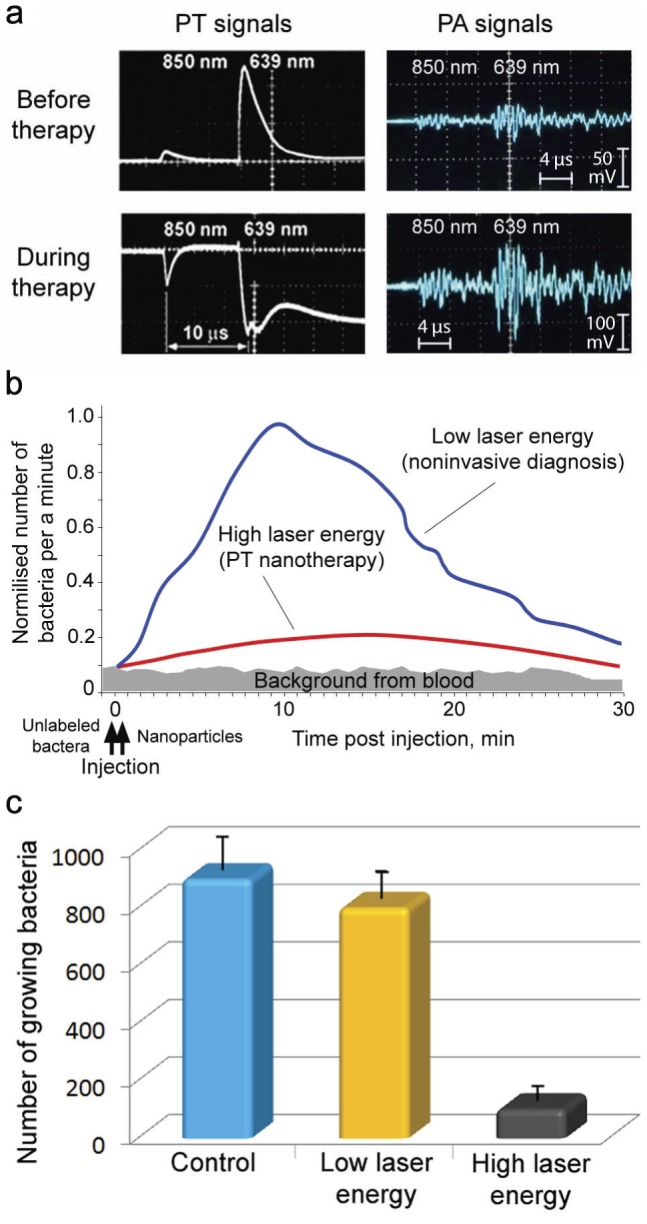
Molecular diagnosis and targeted eradication of *S. aureus* in blood with real-time monitoring of therapeutic efficacy. (**a**) Two-color (639 and 850 nm) monitoring of PT (left) and PA (right) linear (top) and nonlinear (bottom) signals from CBCs labeled with anti-Spa-conjugated siMNPs. (**b**) *In vivo* real-time monitoring of the efficacy of PT nanotherapy of targeted CBCs. The average standard deviation in PA data for each time point at automated data collection was 19%. (**c**) Numbers of viable bacteria in blood samples was determined by plate count as a function of laser irradiation.

PA diagnosis and PT therapy are based on similar physical principles (i.e., laser-induced heat and accompanied acoustic and nanobubble formation) that allow real-time nano-theranostics of CBCs using the same lasers and conjugated NPs. Testing the viability of labeled bacteria *in vitro* with PT and conventional bacteriological assays after a single laser pulse at various energy fluences showed that the threshold photodamage leading to 50% cell death was 0.39±0.16 J/cm^2^ at 900 nm for anti-Spa-conjugated GNR^900^, 0.19±0.11 J/cm^2^ at 671 nm for anti-Lpp-conjugated GNR^690^, 0.51±0.19 J/cm^2^ at 671 nm for anti-Spa-conjugated siMNPs, and 0.08±0.04 J/cm^2^ at 900 nm for anti-Spa-conjugated GNTs^900^. To further test the PA/PT nanotheranostic platform, we performed *in vivo* targeting of CBCs labeled with anti-Spa-conjugated siMNPs using three experimental groups of mice (n = 3). These groups were without laser irradiation (control), PA diagnosis at low laser energy fluence (50 mJ/cm^2^) at 671 nm, and PT therapy by 1-h laser exposure of a 300-µm abdominal blood vessel with laser fluence of 0.8 mJ/cm^2^ at 850 nm and a pulse rate of 10 Hz. Using two-color PAFC/PAFC (640 nm/850 nm) [25] for real-time PA and PT monitoring of blood microvessels allowed for real-time control of noninvasive CBC diagnosis at low laser fluence, CBC killing by strong PT and bubble phenomena at high energy, and monitoring of therapeutic efficiency through the appearance of linear and nonlinear signals (Fig. 9a) and a decreased signal count. PT therapy led to a significant decrease in the PA signal rate (Fig. 9b, red curve) compared to the PA diagnosis group (blue curve) at a laser fluence shown to be safe for blood.

To confirm this therapeutic effect, we repeated this procedure for bacteria labeled *in vitro* prior to injection, showing PT therapeutic effects immediately after injection (Fig. S11) compared to the delayed effects when the bacteria were labeled *in vivo* (Fig. 9b). Mice from all groups, which were labeled *in vivo*, were euthanized and the blood examined for the presence of viable bacteria. Blood from the control and PA diagnostic groups showed similar bacterial growth, while the number of bacteria in blood from the PT therapeutic group was reduced 10 to 12 fold (Fig. 9c). The assessment of collected blood with PT scanning cytometry revealed strong signals from individual bacteria (similar to Fig. 8c) and little or no signals from unbound NPs or their clusters suggesting their negligible levels.

### *In vivo* targeted PT therapy of *S. aureus* in tissue

**Figure 10:**
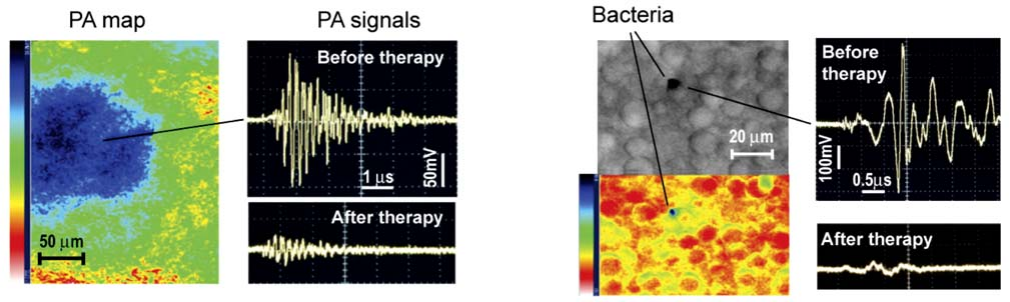
Molecular diagnosis and targeted eradication of *S. aureus* in tissue with real-time monitoring of therapeutic efficacy. (**a**) PA control of targeted PT purging of an infected area in mouse ear (left) by comparison of signals before (right, top) and after (right, bottom) PT therapy at 850 nm (0.8 J/cm^2^). (**b**) Targeted PT therapy of a secondary infection in the liver.

Finally, we demonstrated that theranostic procedure applied to blood could be used for the elimination of bacteria from primary and secondary sites of localized infection *in vivo*. Specifically, the local injection of 10^4^ *S. aureus* cells pre-labeled *in vitro* with anti-Spa-conjugated GNTs into the ear of a mouse demonstrated strong PA signals (Fig. 10a, top right), which completely disappeared after laser exposure (Fig. 10a, bottom right). PA scanning cytometry also allowed us to assess the distribution of bacteria within the injection site before and after PT nanotherapy. Using minimally invasive surgery (small skin incision to provide fiber access to organ), successful PT therapy of a secondary infection site in the liver (Fig. 10b, blue spot in the PA map, bottom left) was also confirmed based on the observation that high-amplitude PA signal levels (top right) returned to the background level of uninfected tissue (bottom right).

## Discussion

The current treatment paradigm for infectious diseases caused by bacterial pathogens is reliant on the use of antimicrobial agents that target metabolic processes unique to prokaryotic cells. This approach, while still a clinical staple, is increasingly compromised by the emergence of pathogens that exhibit resistance to these antibiotics. Indeed, there are no antibiotics currently available to the clinician for which resistance has not been reported, and this includes not only the relatively few agents introduced into clinical practice in the last decade, but in some cases even those currently in the clinical pipeline [51], [52]. This provides direct evidence of the remarkable genetic adaptability of bacterial pathogens, thus making antibiotic resistance essentially an inevitable consequence of the use of any antibiotic. Thus, it is imperative to develop novel therapeutic approaches that are not dependent on the use of such agents.

The nanotechnology-based theranostic platform that we describe here offers many advantages in this respect, the most important being that it is based on the targeted physical destruction of bacterial cells and thus has the potential to retain its therapeutic efficacy irrespective of the antibiotic resistance status of the offending bacteria. This is not to say that resistance could not be developed, one obvious possibility being changes in the targeted surface-associated proteins that alter their immunogenicity to a degree that limits Ab-mediated NP delivery. As with the simultaneous use of multiple Abs, this could potentially be overcome through the use of Ab cocktails similar to those we describe, and one focus of our ongoing work is to identity additional surface-exposed antigens with a specific emphasis on *S. aureus*. Another potentially compromising factor could be the production of capsular polysaccharides to an extent that masks the targeted surface proteins and thereby also limits antibody binding. Indeed, capsule production is an important factor that can compromise antibody and complement mediated opsonization of bacterial cells. Nevertheless, the experiments we describe demonstrate the potential utility of our theranostic approach not only in the context of *S. aureus* but also other bacterial pathogens, the only requirement being identification and characterization of the appropriate bacterial targets. Doing so would also open the door to protocols employing Ab-conjugated NP cocktails targeting different pathogens, thus facilitating the treatment of polymicrobial infections.

In addition to its therapeutic potential in the context of antibiotic resistant strains, the methods we describe would also retain their efficacy even with non-growing or slowly growing bacteria. This is a particularly important consideration with pathogens that cause infections associated with formation of a bacterial biofilm. Indeed, biofilm-associated bacteria exhibit a level of intrinsic resistance that compromises the therapeutic response to essentially all antibiotics [53], thus often necessitating surgical intervention as well as intensive, long-term antimicrobial therapy. As with protocols targeting different bacterial pathogens, this would necessitate the identification of those surface-exposed antigens that define the unique biofilm lifestyle. Indeed, this is one reason we included Abs for the lipoprotein targeted in these studies in that this lipoprotein was identified in a screen of serum from experimentally infected rabbits suffering from biofilm-associated *S. aureus* bone infection [54]. The other reason we included this target is that, like the gene encoding protein A (*spa*), the gene encoding this lipoprotein is highly conserved, and highly expressed, among different strains of *S. aureus* [54]–[56].

Therefore, the potential advantages of the *in vivo* PA/PT nano-theranostic platform can be summarized as follow: (1) physical destruction of bacteria, thus retaining its therapeutic efficacy irrespective of the antibiotic resistance status of the offending bacteria; (2) integration of molecular detection and targeted elimination of fast-moving, 1–2 µm bacteria in heterogeneous blood flow with real-time monitoring of therapeutic efficacy; (3) ultra-high sensitivity (0.5 CFU/mL) for both diagnosis and treatment at the level of a single bacterial cell; (4) the use of NPs as high absorbing, low toxic molecular contrast agents allowing for significant amplification of their signal properties, thus requiring as few as 100–1000 NPs per cell; (5) high spectral specificity based on distinct ultrasharp spectral resonances of gold NPs [27] and asymmetric nonlinear spectra of MNPs (Fig. 6b); (6) intravascular magnetic capturing of CBCs for further amplification and laser ablation; and (7) multiplexing capability using antibody cocktails to avoid the development of resistance and effectively target genotypically and phenotypically diverse strains both within and across different bacterial pathogens, thus facilitating the treatment of polymicrobial infections.

Furthermore, better understanding of the characteristics of life-threatening pathogens is very important for the successful development of antimicrobial therapeutic agents. Surprisingly, many of key questions about bacterial dissemination to distant organs are still unanswered, including migration in tissue, invasion dynamics, interaction with blood and endothelial cells, and extravasations. We believe that the technology we describe here in combination with animal models of bacteremia have established a research tool, which could provide new insights on CBC dissemination, leading to the development of metastatic foci of infection, including endocarditis, osteomyelitis, and infections associated with catheters and implants. Similarly as with the study of CTCs [36]–[40], this tool could facilitate understanding of the CBC properties, including possible re-entering back into the circulation from the distant sites (i.e., lung at pneumonia or brain at meningitis) with further haematogenous spreading to other sites (re-seeding) or even to the site of origin (self-seeding). The presence of small populations of CBCs (in analogy to cancer stem cells), significantly contributing to bacterial colonization and drug resistance, is quite possible.

Finally, PT and PA methods could beneficially supplement each other, and in combination, provide a very powerful diagnostic and therapeutic tool that allows one to verify PA data with more sensitive PTFC in trans-illumination schematic using thin transparent models such as animal ear or mesentery, or to provide PT killing of CBCs with more powerful laser pulses used for PAFC. Because several gold and magnetic NPs were already approved for pilot study on humans [25], and clinical potential and safety of PA technology has been successfully demonstrated in several pilot trials, early laser-based detection and treatment of CBCs could be feasible in a 0.5–1.5 mm hand vein at 1–2 mm depths, which is within well-documented capacity of PA technology to assess much deeper (up to 7 cm) and larger (1 cm) human blood vessels [25], [42], [57], [58]. As an alternative, the bypass schematics (Fig. S12) could be useful for purging of infected blood during sepsis crisis by injection of MNPs at the entrance of bypass and magnetic removal of CBCs and MNPs before connection to systematic circulation. In most of our experiments laser parameters were within laser safe standard (e.g., 60 and 100 mJ/cm^2^ at 900 nm and 1064 nm, respectively) although further optimizations of laser and NP parameters are still needed. The results in this study also hint that the label-free approach at NIR wavelengths could be very useful for rapid (minute-scale), non-toxic and noninvasive diagnosis of skin infections, and potentially for disinfection of instruments or food without antimicrobial agents.

## Methods

### Integrated PT and PA techniques

Laser-induced PT, PA and bubble-formation phenomena in samples were evaluated with an integrated PT/PA microscope as described in detail elsewhere [25], [27], [40]. In brief, the first setup was constructed on the technical platform of an upright Olympus BX51 microscope (Olympus America). A tunable optical parametric oscillator (OPO) (Lotis Ltd) was used to irradiate samples with the following parameters: wavelength, 420–2,300 nm; pulse width, 8 ns; beam diameter, 10–50 mm; fluence range, 1–10^4^ mJ/cm^2^; pulse repetition rate, 10 Hz. Second setup was built on the technical platform of an inverted microscope (IX81, Olympus America.,USA), and a tunable OPO (Opolette HR 355 LD, OPOTEK, Inc., Carlsbad, CA) with the following parameters: spectral range of 410–2500 nm, pulse width, 5 ns; pulse energy, up to 5 mJ, pulse energy stability, 3–5%; pulse repetition rate, 100 Hz; line width ∼0.5 nm. The setup was equipped also with three high-pulse-repetition-rate nanosecond lasers with the following parameters: 1) wavelength, 671 nm; pulse energy, 35 µJ; pulse width, 25 ns; and repetitions rate, up to 30 kHz (model: model: QL671-500, CrystaLaser, Reno, NV, USA); 2) 820 nm; 75 µJ, 8 ns, and 30 kHz (model: LUCE 820, Bright Solutions); and 3) 1,064 nm, 100 µJ, 10 ns, and 500 kHz (model: MOPA-M-10, Multiwave Photonics, Porto, Portugal). Laser-induced PA waves were detected with an ultrasound transducer (XMS-310, 10 MHz; Panametrics) that was gently attached to the slide or tissue. Warm water or ultrasound gel was applied for better acoustic matching between the transducer and the samples. After amplification (Panametrics amplifier model #5662; bandwidth, 50 kHz to 5 MHz; gain, 60 dB), signals were recorded by either a computer or a Tektronix TDS 3032B oscilloscope, and then analyzed with standard and customized software. The PA signals had a bipolar shape that was transformed into a pulse train due to reflection effects. In PT thermal lens mode, laser-induced temperature-dependent variations of the refractive index around absorbing zones caused defocusing of a continuous- wave collinear He-Ne laser (model 117A, Spectra-Physics, Inc.; wavelength, 633 nm; 1.4 mW) probe beam, leading to a reduction in the beam's intensity at its center, detected by a photodiode with preamplifier (PDA36A, 40 dB amplification, ThorLabs Inc.). The linear PT thermolens signals demonstrated the standard fast-rising unipolar positive peak associated with rapid (picosecond-nanosecond scale) sample heating and a slower (microsecond scale) tail corresponding to sample cooling. Lased-induced nanobubbles around overheated targets (e.g., chromophores or nanoparticles) lead to appearance of nonlinear negative peak due to scattering and refraction of probe beam. PT/PA mapping of the sample (e.g., bacteria in the blood) was realized by sample-scanning with two-dimensional (X-Y) automatic translation stage (Conix Research, Inc. in BX-51 and H117 ProScan II, Prior Scientific, Inc. in IX-81). Some samples were examined in a chamber (S-24737, Molecular Probes) with thickness of ∼120 µm or in a slide with coverglass with thickness of 1–2 µm.

Laser radiation was delivered to samples by using a customized condenser to create either circular beam (the lateral resolution was ∼0.7 µm with a 20× objective, and a ∼300 nm with 100× oil immersion objective) or linear beam shapes whose dimensions could be adjusted from 10×50 µm to 25×100 µm by positioning the axial condenser. As an alternative, a fiber with miniature cylindrical optics was used to deliver laser radiation directly to skin vessels (*e.g.*, for PT therapy). The PT setups were equipped with a high-speed (200 MHz) analog-to-digital converter board (National Instruments Corp., PCI-5152, 12-bit card, 128 MB of memory), specialized software (LabVIEW; National Instruments), and a Dell Precision 690 workstation with a quadcore processor, 4 GB of RAM and Windows Vista 64-bit operating system. The PA signals generated by high pulse repetition rate lasers were recorded, digitized (14-bit resolution, 125 mega-samples per second; model: custom AD484; 4DSP Inc., Reno, NV), and analyzed with custom-written software on the workstation (Precision T7500, Dell, Round Rock, TX). Each trace is analyzed for the presence of peaks with height exceeding a defined threshold height. The stable baseline due to the background absorption by blood is subtracted electronically and accurately, that helps to observe very small peaks.

### High-resolution and high-speed transmission and fluorescent microscopy

The PA and PT microscopes were also equipped with high resolution fluorescent and transmission modules. A cooled, color CCD camera (DP42, Olympus) that was used for the navigation of laser beams and for the verification of PA and PT data with fluorescent images using specific fluorescent tags. High-resolution (300 nm) transmission digital module (TDM) fitted with a high-speed (up to 40,000 fps) CMOS (MV-D1024-160-CL8; Photonfocus AG, Lachen, Switzerland) and high sensitive CCD (Cascade: 512; Photometrics, Roper Scientific, Inc.) cameras was used for imaging blood vessels, ear and other organs with bacteria.

### Absorption Spectroscopy

The optical absorption spectra of *S. aureus* suspension in PBS (10^8^ cells/1 mL) were examined using a fiber spectrophotometer (USB4000, Ocean Optics Inc, USA).

### Magnetic module

The local permanent magnetic field in selected vessels was provided by a cylindrical Neodymium-Iron- Boron (NdFeB) magnet with Ni-Cu-Ni coating, 3.2 mm in diameter and 9.5 mm long with a surface field strength of 0.39 Tesla (MAGCRAFT, Vienna, VA).

### Nanoparticles

Eight different NP types were explored in this study: GNTs, 30-nm MNPs without and with silica layer (siMNPs), 50-nm MNPs, PEG-coated GNRs with maximal absorption at 690 nm and 900 nm and GNRs at 725 nm and 900 nm without polyethylene glycol (PEG). GNTs with an average size of ∼12×98 nm and maximal absorption at 850–1000 nm were synthesized as described elsewhere with detailed reports on their physicochemical characteristics [42]. The GNRs with maximal absorption at 690 were purchased from Nanopartz Inc. (Loveland, CO). GNRs with maximal absorption of 900 nm with and without PEG coating. The superparamagnetic iron oxide MNPs consisted of a magnetite (Fe_3_O_4_) ∼50-nm core (3 stands for 3 crystals of 10–15 nm each) were coated with 10-nm dextran layer (*i.e.*, the total MNP size was ∼70 nm) and FITC covalently attached to activated sites on the Dextran (Clementer Associates, Madison, CT). The 30-nm MNP with thin layer of carboxylic acid, of -COOH in original concentration of 2.4×10^12^/mL were from Ocean Nanotechnology. These MNPs were coated with 10-nm silica-layer (si-MNPs). Selected nanoparticles were additionally conjugated with Fluorescein Isothiocyanate (FITC).

The GNTs and MNPs were conjugated with Ig fraction of anti-protein A (anti-Spa; Rockland Inc., Gilbertsville, PA) according to the manufacturer's specifications. GNRs with maximal absorption at 900 nm (GNR^900^) bioconjugated with anti-Spa antibodies and GNRs with maximal absorption at 690 nm (GNR^690^) bioconjugated with anti-lipoprotein antibodies (anti-LPP; kindly provided by Dr. Mark Shirtliff) were purchased from Nanopartz Inc. (Loveland, CO). The FITC-conjugated Ig fraction of anti-Spa (Rockland Inc., Gilbertsville, PA) and Phycoerythrin (PE)-conjugated anti-Lpp antibodies were additionally purchased for verification by fluorescence imaging taking into account the excitation of 490 nm and emission of 525 nm for FITC and the excitation of 488 and 565 nm and emission of 578 nm for PE.

### Atomic force and transmission electron microscopy

The physical characteristics of nanoparticles were assessed using AFM and TEM as reported elsewhere [42], [59]. Briefly, the AFM imaging was carried out with Veeco Multimode Scanning Probe Microscope with Nanoscope IIIa Controller (Veeco Instruments, Woodbury, NY). For AFM sample preparation, 25 µL of each sample solution was mixed with 25 µL of DI water and 5 µL of the mixture was dispensed on a mica substrate (Novascan, Ames, IA). All samples were scanned in tapping mode in air with a NanoWorld Pointprobe® NCSTR AFM probe (NanoWorld AG, Neuchâtel, Switzerland), which is designed for soft tapping mode imaging and enables stable and accurate measurements with reduced tip-sample interaction, in order to obtain high-resolution AFM images with minimal sample damage. The sample scan rate was 1.0 Hz with an aspect ratio of 1∶1. The force constant of the tip for scanning was 7.4 N/m. The free resonance frequency of the cantilever was automatically tuned by the Nanoscope Software (version v5.31r1; Veeco Instruments). For TEM studies, 1.5 µL of each sample solution was pipetted on a 300 mesh Formvar-coated copper grid, followed by drying in the air. The TEM samples were examined using either a JEOL 100 CX Transmission Electron Microscope at 100 keV or FEI Titan 80–300 S/TEM fitted with an image corrector, an X-ray energy dispersive detector (EDAX) and a post-column energy filter (GIF from Gatan).

### Bacterial cells

*S. aureus* strain designated UAMS-1 was isolated from a patient with osteomyelitis at the McClellan Veterans Hospital in Little Rock, Arkansas [30]. The strain was deposited with the American Type Culture Collection and is available as strain ATCC 49230. Bacteria were cultured according to standard procedure.

Bacteria were incubated with nonconjugated nanoparticless at 37C° for 30 and 60 minutes and with functionalized NPs at 37C° for 30 minutes through specific binding. The resultant bacterium–NP complexes were washed three times in PBS by centrifugation (5,000 rpm; 3–5 min). Both, conjugated and non-conjugated cells were typically incubated at the relation of 1,000–10,000 NPs per one bacterium. In selected tests with anti-Spa-GNTs and anti-Spa-GNRs we used proportions ranged from 10 NPs to 100,000 NPs per one bacterium. The attachment of NPs to bacterial cells was verified by three ways: (1) comparing PT signals from individual labeled and unlabeled cells at appropriate wavelength coinciding with maximum absorption of nanoparticles; (2) imaging with AFM or TEM; and (3) fluorescent imaging when NPs were additionally labeled with fluorescent dyes.

In case of targeting bacteria with MNPs the magnetic capture of bacteria were studied by the attachment of magnet above the sample. Viability of *S. aureus* after each procedure was assessed by plating on trypic soy agar with antibiotic selection.

### Blood samples

To verify labeling efficiency in the complex environment of blood circulation, stabilized blood samples (10 µL) from donor mice and rats were spiked with 10^4^ bacterial cells resuspended in 1 µL of PBS. Unlabeled *S. aureus*, bacteria labeled with Abs alone, bacteria labeled with non-conjugated GNTs, MNPs, and GNRs, or bacteria targeted by functionalized anti-Spa-GNTs, anti-Spa-MNPs, anti-Spa-GNRs^900^ and anti-lipoprotein-GNRs^690^ as well as by cocktail of NPs (anti-Spa-GNRs^900^+anti-lipoprotein-GNRs^690^ in equal proportions) were used in these tests. High-resolution TDM imaging was used to image bacteria in thin slide while additional labeling bacteria with FITC was applied to identify bacteria in thick slide with fluorescent microscopy.

Direct labeling in blood was achieved when cocktail of nanoparticles (10^9^ anti-Spa-GNRs^900^ in 1 µL of PBS+10^9^ of anti-lipoprotein-GNRs^690^ in 1 µL of PBS) and bacteria (10^5^ bacteria in 5 µL of PBS) were added to the 100-µL of intact blood and incubated at 37°C for 30 minutes in incubator with shaker.

### Animal models

All animal work was approved by the University of Arkansas for Medical Sciences Institutional Animal Care and Use Committee. Nude mice nu/nu weighing 20–25 g and rat weighing 150–200 g (both from Harlan Sprague–Dawley) were used in accordance with protocols approved by the University of Arkansas for Medical Sciences Institutional Animal Care and Use Committee. Most experiments were performed (1) on well-distinguished mouse ear blood vessels with a diameter of 50–100 mm and blood velocities of 3–7 mm/s, (2) on larger blood vessels with a diameter of 200–300 mm in the abdominal area of the mouse at a depth of 0.3–0.5 mm and flow velocity of 1–3 cm/s, and (3) on mesenteric blood vessels [48]. Selected experiments were performed also with rat ear, which still had a distinguishable blood microvessel, in spite of its thicker structure compared to mice ear, and with the rat mesentery. The mesentery has an almost ideal biostructure for verification of PA and PT data *in vivo* because it consists of very thin (10–15 µm) transparent connective tissue with a single layer of blood and lymph microvessels assessable with optical technique in transmission schematics. After standard anesthesia, mice were placed on the heated microscope stage with a topical application of warm water on ear or skin fold for acoustic matching of the ultrasound transducer and the tissue. A small surgery was applied for mesenteric model but the preparation procedure was safe and did not affect cell properties for at least 5 h. After induction of anesthesia (ketamine/xylazine, 50/10 mg/kg, i.m. for mice, and ketamine/xylazine, 60/15 mg/kg for a rats) animals were laparotomized by a midabdominal incision, and the small-intestinal mesentery was placed on a customized, heated (37.7°C) microscope stage and suffused with warmed Ringer's solution (37°C, pH 7.4) containing 1% bovine serum albumin to prevent protein loss.

The capacity PA and PT theranostic platform were estimated by introduction of NPs using intravenous injection into the animal tail vein, by intracardiac injection or through catherter in jugular vein. The experimental design included injections of various NPs in concentration ranged from 10^9^ to 10^11^ nanoparticles in 30 µL of PBS.

The local staphylococcal infection was modeled by subcutaneous injection of 20 µL (10^4^ colony-forming-unit (CFU)/mL) of bacterial suspension in mouse ear tissue. Staphylococcal bacteremia was produced by inoculation of 10^5^ (for mice) to 10^7^ (for rats) bacterial cells in 40–50 µL of PBS. The secondary infectious sites were diagnosed with PA scanning cytometry of bones (tibia), liver and spleen in 3 hrs and 2 days after inoculation. After euthanasia organs were immediately gentle extracted, placed on slide with few-mm thick chamber and *ex tempore* scanned.

After injection of MNPs or bacteria labeled with MNPs the magnetic capturing cells was investigated by gentle attachment of magnet close to detection site. The distances between the magnet and examined vessels were ranged from 50 to 100 µm for ear vessels; 0.3 to 0.5 mm for abdominal vessels and 10–20 µm for mesenteric vessels.

Occasionally strong PA signals were observed after intravenous injection of GNRs during one minute, which were associated with NP aggregates, which were quickly (10–20 minutes) cleared from circulation compared to individual NPs. To minimize this effect, before experiments, NP clusters were disaggregated by ultrasound and then filtered. If still few unpredictable signals occurred immediately after injection, they did not influence signals related with CBC targeting *in vivo* because later was coming with significant time delay (>5 minutes).

The cytotoxicity related to bacterial cells and host cells was, first, assessed *in vitro* by monitoring cell viability and proliferation for 10 days [42]. The *in vivo* cytotoxicity was evaluated by testing of multiple organs after exposure to GNRs at concentrations as high as 0.5 mg/ml for as long as 10 days. Based on previous and current toxicity tests GNRs without PEG layers were excluded from further study due to high toxicity.

Intravenous injection in tail and in large jugular vein through catheter, or intracardiac injection showed similar bacteria clearance time. However, administration through heart and jugular vein provided fastest bacteria appearance in ear vessels (few seconds) with maximal CBC rate, while maximal delay in bacterial arrival and minimum rate occurred after tail injection due to longer blood network and profound bacteria arrests in pulmonary microvessels

### Statistical analysis

A minimum of 3 animals was used for each experiment unless otherwise noted. Results are expressed as mean ± standard error. Spearman correlations for which P<0.05 were considered statistically significant. MATLAB 7.0.1 (MathWorks, Natick, MA), and LabVIEW (National Instruments) were used for the statistical calculations. Data was summarized as the mean, standard deviation (SD), median, inter-quartile range, and full range. Comparisons of PA and PT data were via scatter plot in conjunction with Spearman correlation analysis.

## Supporting Information

Figure S1 **Schematics of PA and PT flow cytometer/microscope.**
(TIF)

Figure S2 **Light microscopy and atomic force microscopy (AFM) images of golden carbon nanotubes (GNTs) alone and on the surface of** ***S. aureus*** **cells.** (**a**) Epi-fluorescence and light microscopy images after incubation with fluorescein-labeled anti-Spa Ab conjugated with GNTs. Inset is the magnified light microscopy image of the dash-box region in the fluorescence image. The images in white boxes (scale bar, 0.5 µm) are magnified images of *S. aureus* cells (dash circles). Topographic AFM images of a single *S. aureus* cell before (**b**) and after (**c**) targeting by anti-Spa conjugated GNTs. Arrows in panel **c** indicate surface-localized GNTs, while insets are the surface plots (45° view) of magnified topographic images (dash boxes in the main image). Targeting efficiency of anti-Spa Ab to *S. aureus* in PBS was 96.3% (±4.23%) on the basis of the light microscopy image analyses. Specifically, the epi-fluorescence images were compared with the counterpart light microscopy image as exemplified in panel **a**. The number of GNTs bound to *S. aureus*, on the basis of the image analyses at multiple sections of the GNT-targeted cells, varied from 50 to 400 per cell (∼100 GNTs per cell on average) depending on incubation time and concentration of bacteria and GNTs, with the image shown in panel **a** being obtained using ∼10^6^ CFU/mL *S. aureus* and ∼10^8^/mL GNTs.
(TIF)

Figure S3 **Transmission electron microscopy (TEM) images of silica-coated iron oxide magnetic nanoparticles (siMNP).** Scale bars, 100 nm (panels, **a** and **b**) and 10 nm (panels, **c–g**). Silica coating efficiency was ∼100%. The majority of siMNPs contained either one MNP core (panels, **e–g**) or two or more MNP cores (MNP clusters) (panels, **c** and **d**). The thickness of silica coating was ∼10 nm on average for the siMNP with single MNP core.
(TIF)

Figure S4 **Absorption spectra of nanoparticles (NPs).** CNT, carbon nanotubes; GNS, gold nanoshells; GNT, golden carbon nanotubes; GNR, gold nanorods; MNP, magnetic nanoparticles; and ICG, Indocyanin Green dye.
(TIF)

Figure S5 **Optical images of** ***S. aureus*** **with and without NPs.** Images were obtained at the specified magnification with no NPs (control) or after incubations with nonconjugated NPs. Incubation was performed for 30 min at 37°C using 10^5^ bacteria and 10^8^ each nanoparticles.
(TIF)

Figure S6 **Average cluster sizes of NPs around individual bacteria or small aggregates of bacteria.**
(TIF)

Figure S7 **Typical levels of laser energy fluence producing detectable PT signals from** ***S. aureus*** **cells labeled with non-conjugated NPs (GNTs, GNRs, GNSs, and MNPs) and dye (ICG).**
(TIF)

Figure S8 (**a**) Experimental schematics for laser-based detection of bacteria in blood *ex vivo*. Typical photothermal (PT) (top) and photoacoustic (PA) (bottom) signals from blood alone (**b**) and from labeled individual bacteria in blood (**c**).
(TIF)

Figure S9 **Optical (a) and fluorescent images (b,c) of individual** ***S. aureus*** **cells labeled with conventional dyes without antibodies**: FITC (**b, green**) and PE (**c, red**). The labeling was used to identify bacteria in animal tissues and blood.
(TIF)

Figure S10 **Histology of infected blood.** Approximately 1000 bacteria were injected into the rat tail vein. Blood was drawn and subjected to PT mapping (**a**) and histological evaluation (**b**). Comparison of PT and histological data were used to estimate a sensitivity threshold of 0.5 CFU/mL. The images on top and bottom were obtained from the same blood sample with different magnification.
(TIF)

Figure S11 ***In vivo*** **eradication of** ***S. aureus*** **priory labeled** ***in vitro*** **with real-time monitoring of therapeutic efficacy.**
(TIF)

Figure S12 **Principle and animal model for** ***in vivo*** **PA detection, molecular targeting, and PT purging of infected blood with an extracorporeal schematic.** Catheters are placed in a large artery or vein (*e.g.*, jugular) to create a bypass. The siMNPs, conjugated with Ab against staphylococcal protein A (Spa), were injected into a tube. The distance between the injection site and detection points could vary by changing the tube length. Cells labeled in flow were captured by the magnet. Laser irradiation of the area near the magnet generates PA signals, which were detected with an ultrasound transducer attached to the tube. Simultaneously, laser irradiation at higher energy allowed PT killing of targeted bacteria. Conventional transmission imaging made it possible to simultaneously control the positions of the laser beam, magnet, and transducer. High-speed imaging also allowed visualization of individual moving cells at the single-cell level as shown in the images (right, bottom) obtained at different magnifications (4×, 20×, and 100×, respectively). Thus, the extracorporeal (bypass) schematic could provide continuous PA monitoring of blood flow in external tubes, and permit efficient capture of abnormal objects (*e.g.*, bacteria or its toxins targeted by MNPs directly in the extracorporeal flow). Magnetic capture of both abnormal objects and unbound magnetic nanoparticles prevents them from being transported further in the systemic circulation.
(TIF)
