# Supplementary figures and images for: In Vivo Magnetic Enrichment, Photoacoustic Diagnosis, and Photothermal Purging of Infected Blood Using Multifunctional Gold and Magnetic Nanoparticles
Source: PLoS One. 2012 Sep 26;7(9):e45557. doi: 10.1371/journal.pone.0045557 (PMC3458934; doi:10.1371/journal.pone.0045557)

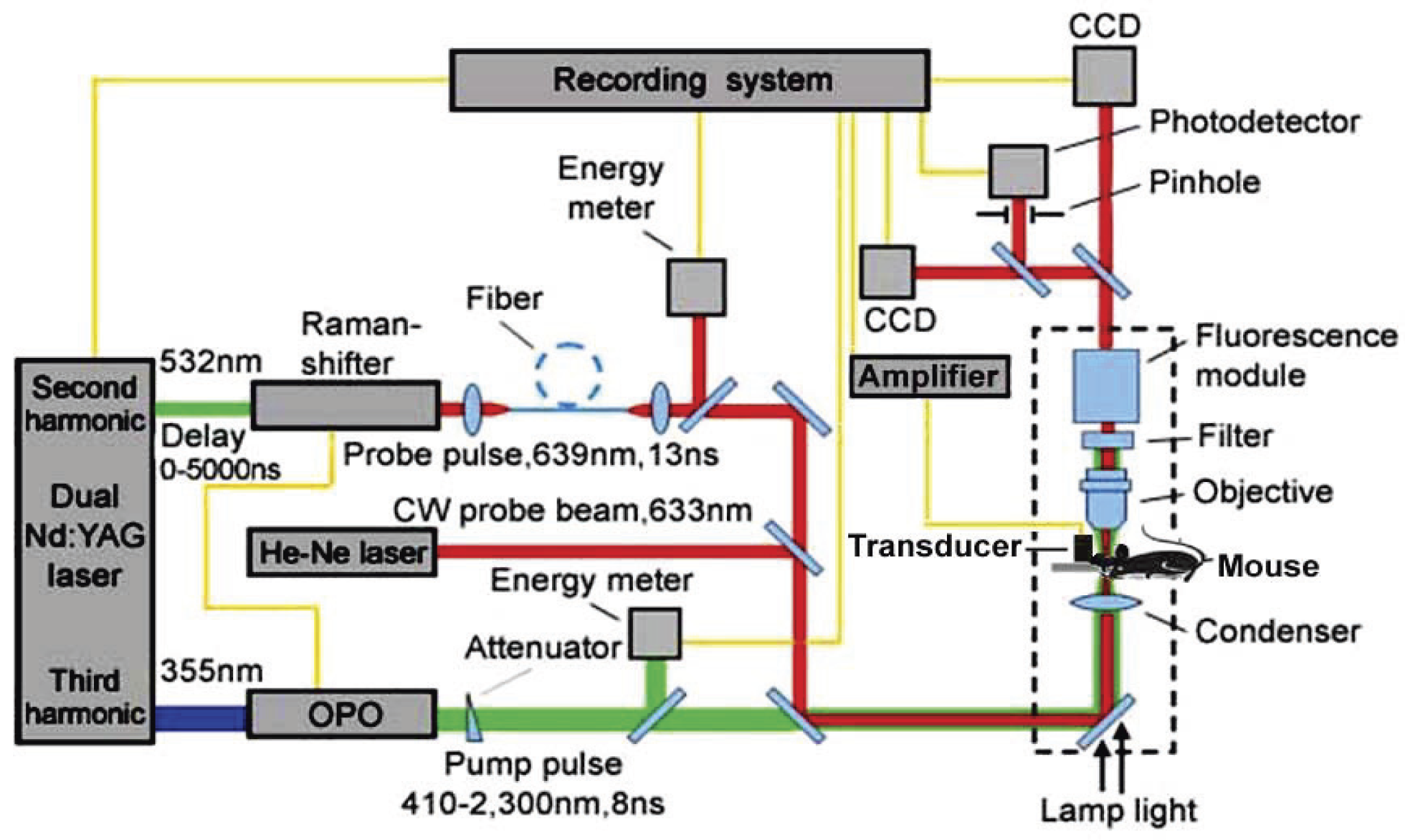

Supplement: Figure S1 — Schematics of PA and PT flow cytometer/microscope. (TIF) [file pone.0045557.s001.tif]

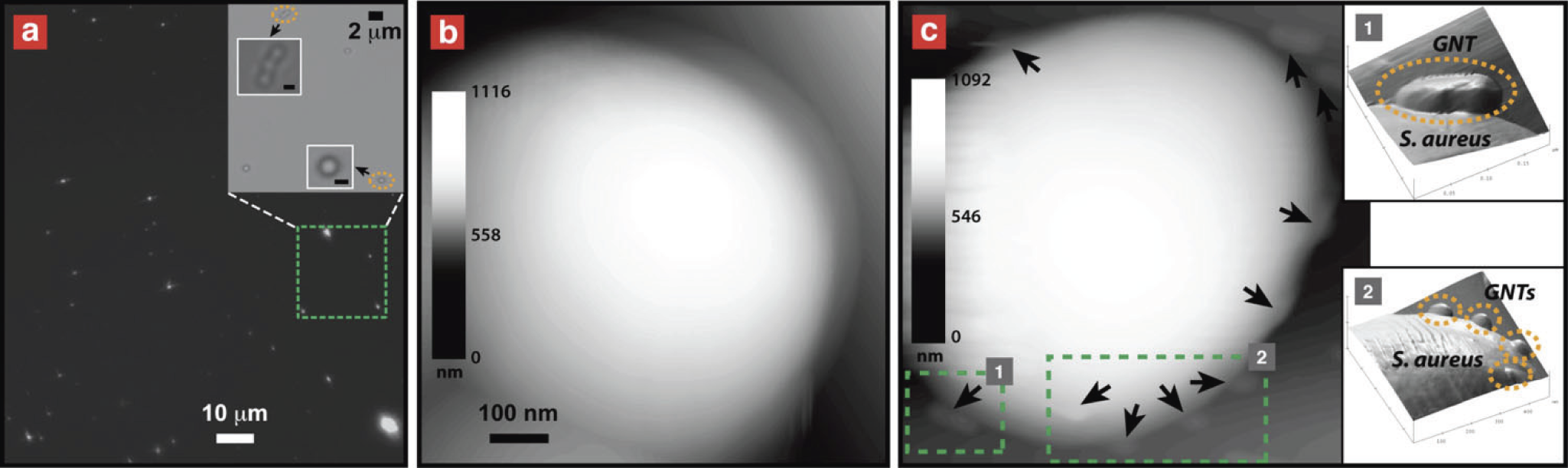

Supplement: Figure S2 — Light microscopy and atomic force microscopy (AFM) images of golden carbon nanotubes (GNTs) alone and on the surface of S. aureus cells. (a) Epi-fluorescence and light microscopy images after incubation with fluorescein-labeled anti-Spa Ab conjugated with GNTs. Inset is the magnified light microscopy image of the dash-box region in the fluorescence image. The images in white boxes (scale bar, 0.5 µm) are magnified images of S. aureus cells (dash circles). Topographic AFM images of a single S. aureus cell before (b) and after (c) targeting by anti-Spa conjugated GNTs. Arrows in panel c indicate surface-localized GNTs, while insets are the surface plots (45° view) of magnified topographic images (dash boxes in the main image). Targeting efficiency of anti-Spa Ab to S. aureus in PBS was 96.3% (±4.23%) on the basis of the light microscopy image analyses. Specifically, the epi-fluorescence images were compared with the counterpart light microscopy image as exemplified in panel a. The number of GNTs bound to S. aureus, on the basis of the image analyses at multiple sections of the GNT-targeted cells, varied from 50 to 400 per cell (∼100 GNTs per cell on average) depending on incubation time and concentration of bacteria and GNTs, with the image shown in panel a being obtained using ∼106 CFU/mL S. aureus and ∼108/mL GNTs. (TIF) [file pone.0045557.s002.tif]

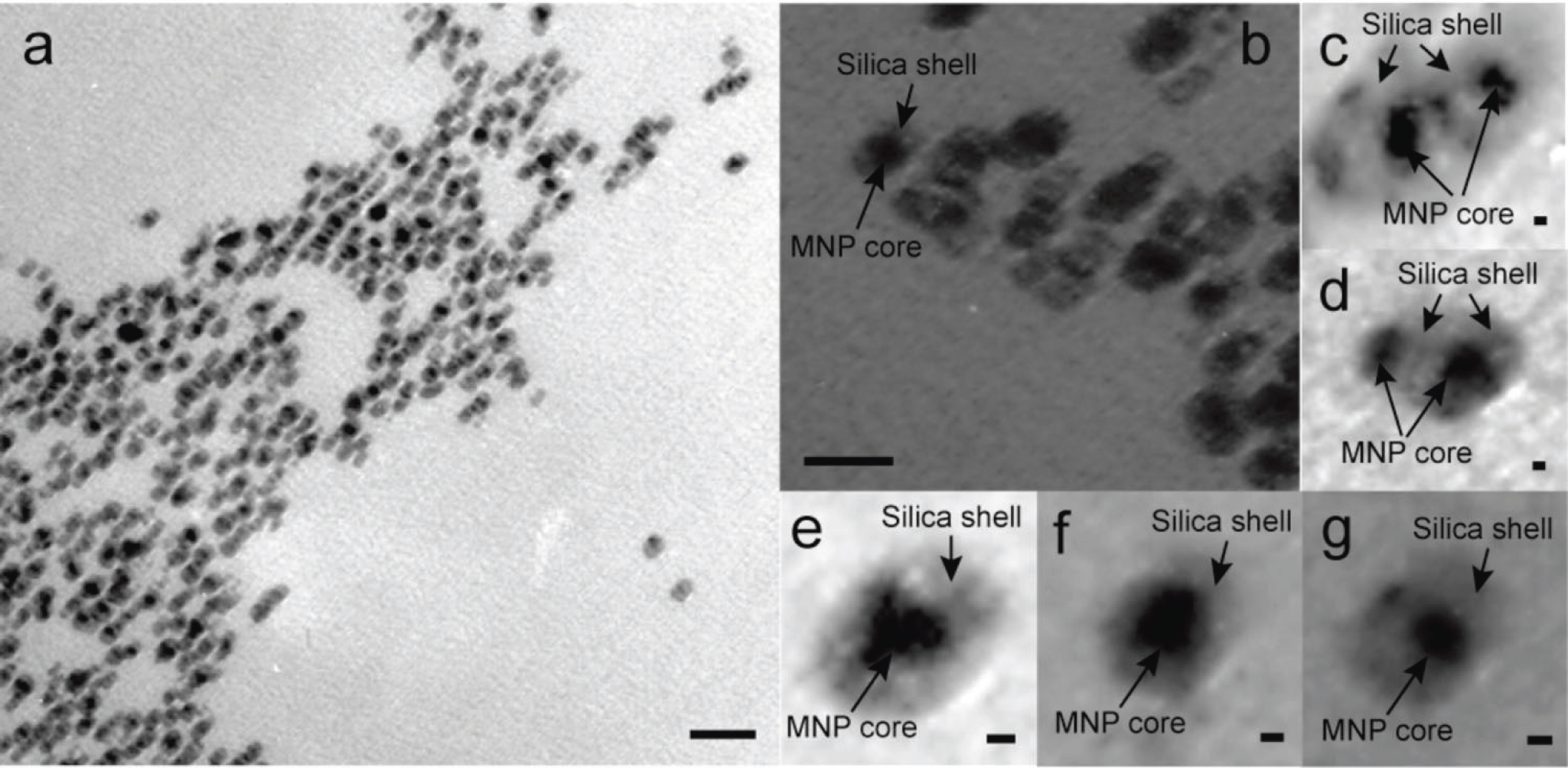

Supplement: Figure S3 — Transmission electron microscopy (TEM) images of silica-coated iron oxide magnetic nanoparticles (siMNP). Scale bars, 100 nm (panels, a and b) and 10 nm (panels, c–g). Silica coating efficiency was ∼100%. The majority of siMNPs contained either one MNP core (panels, e–g) or two or more MNP cores (MNP clusters) (panels, c and d). The thickness of silica coating was ∼10 nm on average for the siMNP with single MNP core. (TIF) [file pone.0045557.s003.tif]

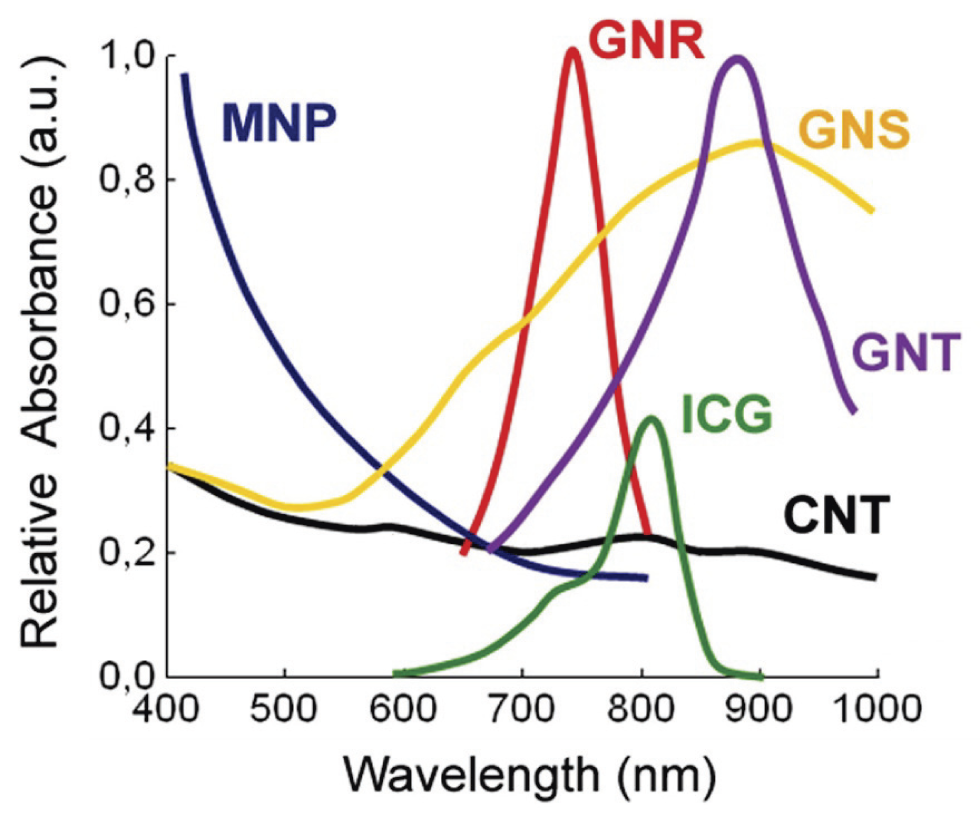

Supplement: Figure S4 — Absorption spectra of nanoparticles (NPs). CNT, carbon nanotubes; GNS, gold nanoshells; GNT, golden carbon nanotubes; GNR, gold nanorods; MNP, magnetic nanoparticles; and ICG, Indocyanin Green dye. (TIF) [file pone.0045557.s004.tif]

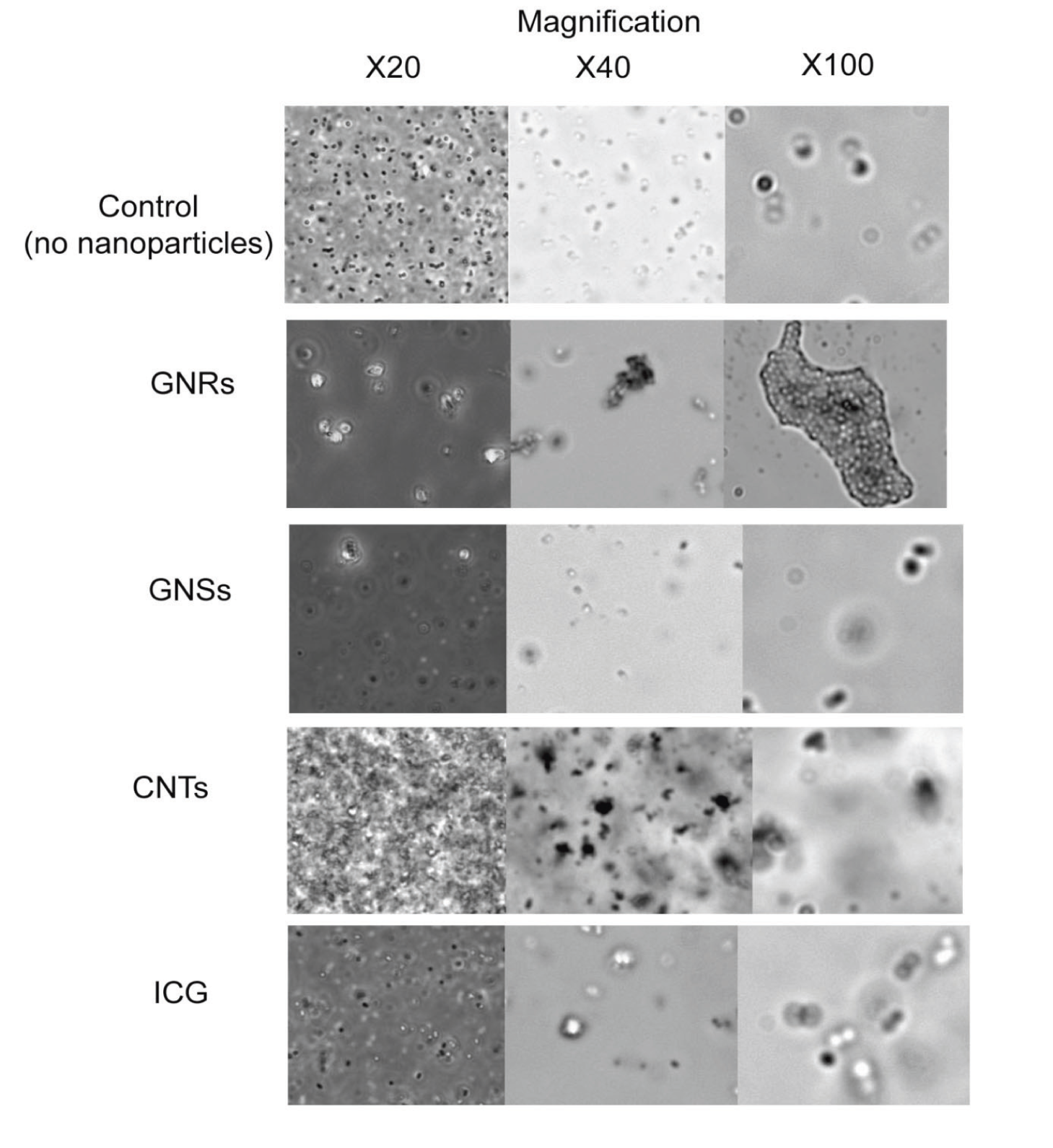

Supplement: Figure S5 — Optical images of S. aureus with and without NPs. Images were obtained at the specified magnification with no NPs (control) or after incubations with nonconjugated NPs. Incubation was performed for 30 min at 37°C using 105 bacteria and 108 each nanoparticles. (TIF) [file pone.0045557.s005.tif]

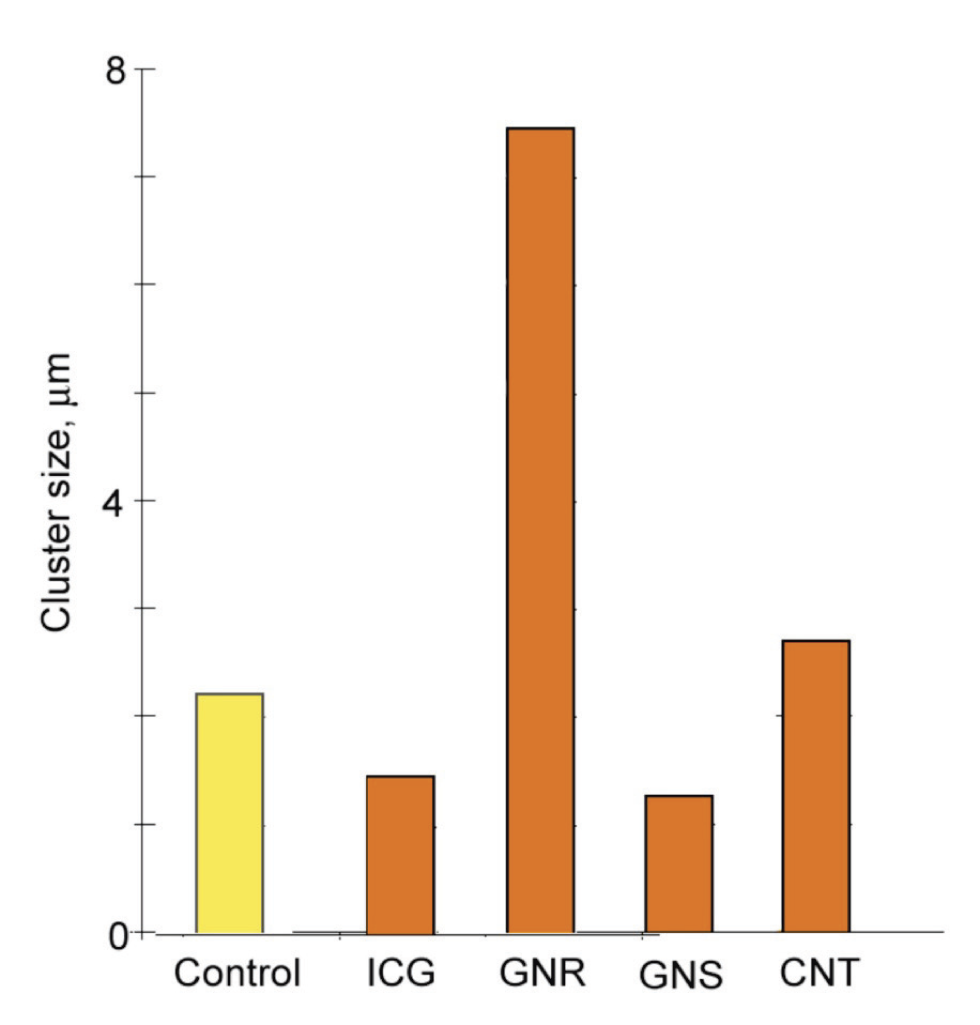

Supplement: Figure S6 — Average cluster sizes of NPs around individual bacteria or small aggregates of bacteria. (TIF) [file pone.0045557.s006.tif]

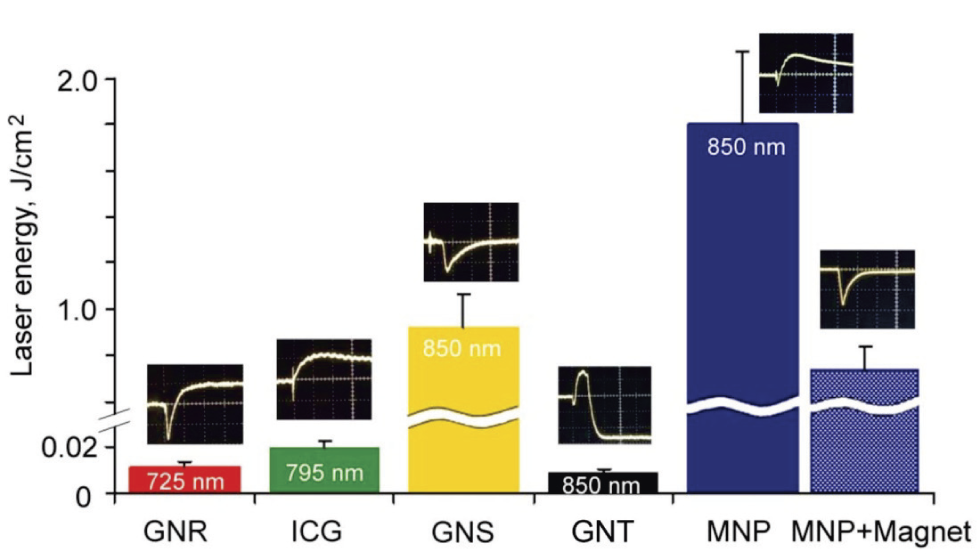

Supplement: Figure S7 — Typical levels of laser energy fluence producing detectable PT signals from S. aureus cells labeled with non-conjugated NPs (GNTs, GNRs, GNSs, and MNPs) and dye (ICG). (TIF) [file pone.0045557.s007.tif]

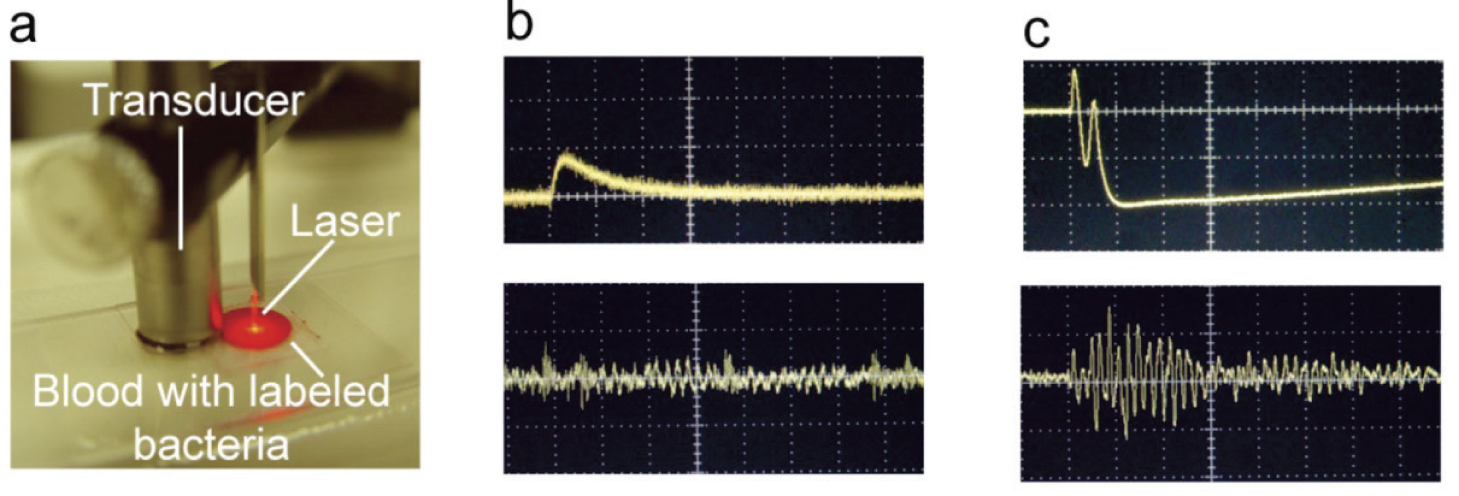

Supplement: Figure S8 — (a) Experimental schematics for laser-based detection of bacteria in blood ex vivo. Typical photothermal (PT) (top) and photoacoustic (PA) (bottom) signals from blood alone (b) and from labeled individual bacteria in blood (c). (TIF) [file pone.0045557.s008.tif]

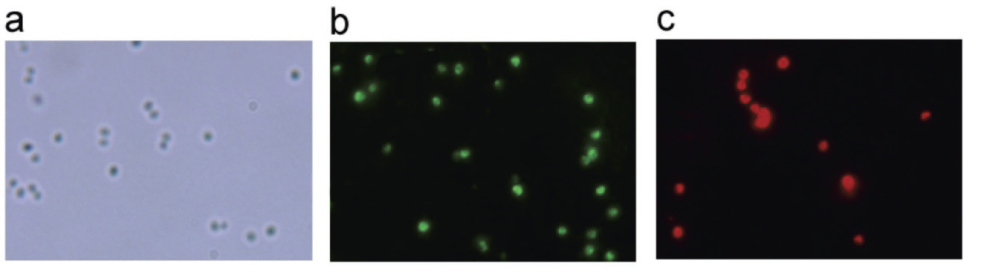

Supplement: Figure S9 — Optical (a) and fluorescent images (b,c) of individual S. aureus cells labeled with conventional dyes without antibodies: FITC (b, green) and PE (c, red). The labeling was used to identify bacteria in animal tissues and blood. (TIF) [file pone.0045557.s009.tif]

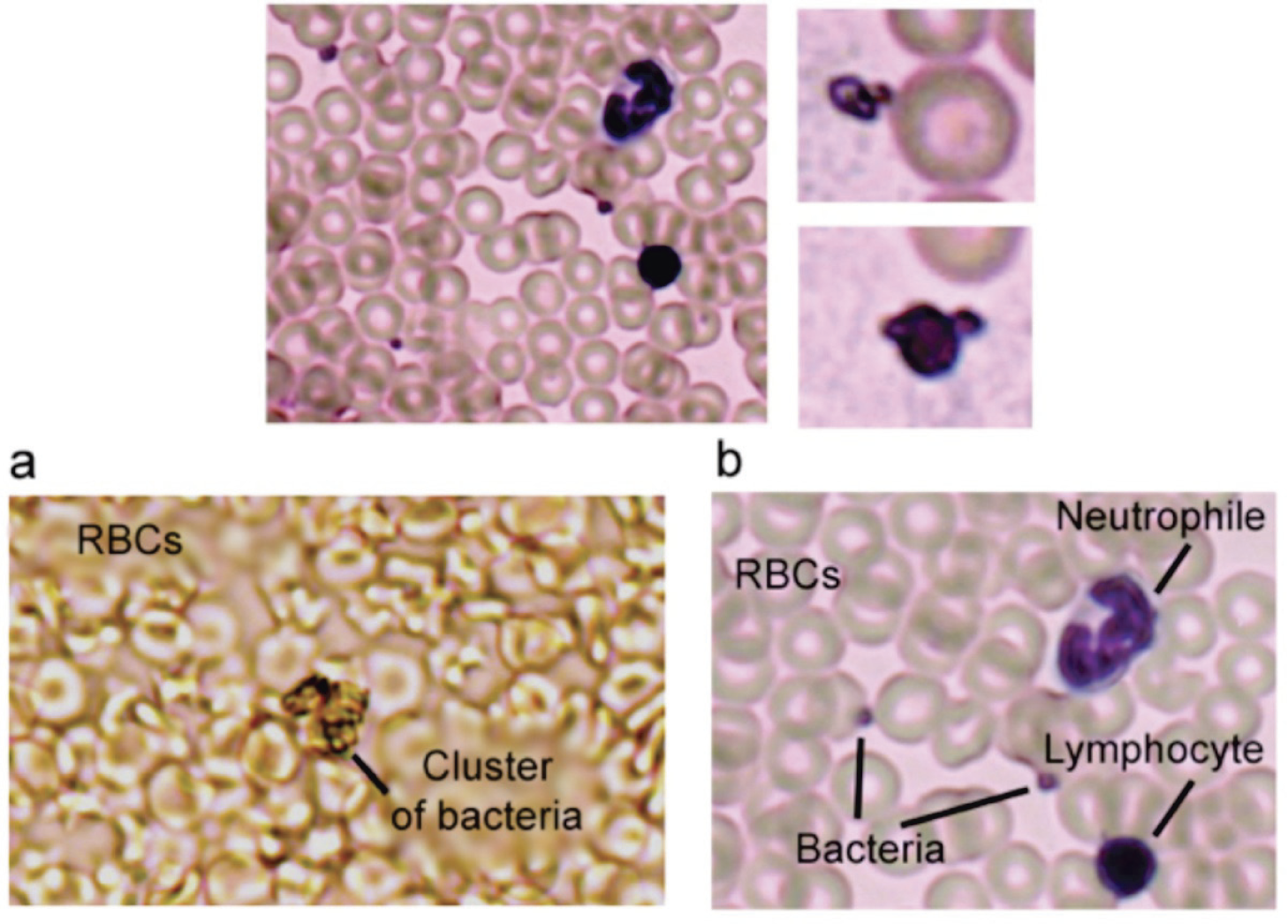

Supplement: Figure S10 — Histology of infected blood. Approximately 1000 bacteria were injected into the rat tail vein. Blood was drawn and subjected to PT mapping (a) and histological evaluation (b). Comparison of PT and histological data were used to estimate a sensitivity threshold of 0.5 CFU/mL. The images on top and bottom were obtained from the same blood sample with different magnification. (TIF) [file pone.0045557.s010.tif]

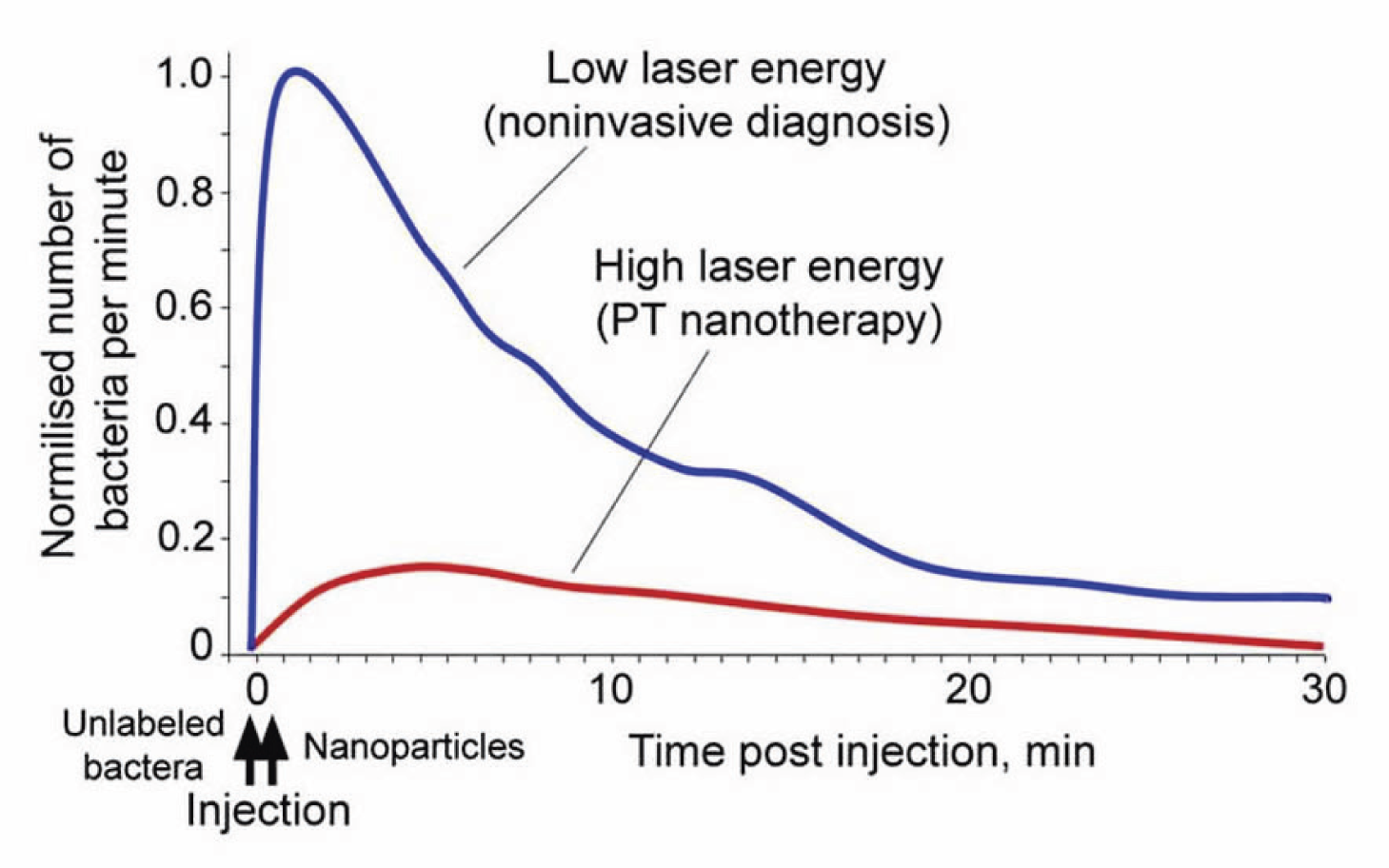

Supplement: Figure S11 — In vivo eradication of S. aureus priory labeled in vitro with real-time monitoring of therapeutic efficacy. (TIF) [file pone.0045557.s011.tif]

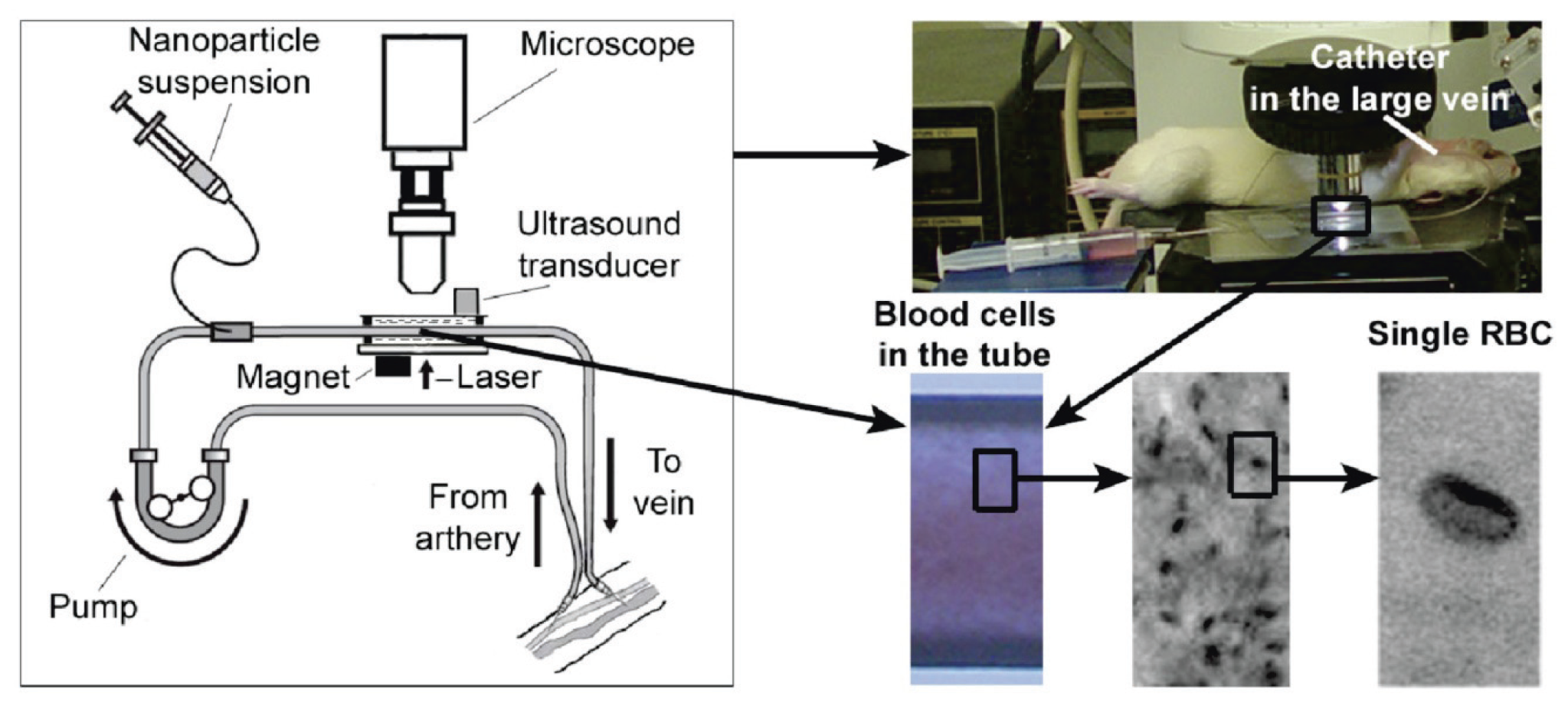

Supplement: Figure S12 — Principle and animal model for in vivo PA detection, molecular targeting, and PT purging of infected blood with an extracorporeal schematic. Catheters are placed in a large artery or vein (e.g., jugular) to create a bypass. The siMNPs, conjugated with Ab against staphylococcal protein A (Spa), were injected into a tube. The distance between the injection site and detection points could vary by changing the tube length. Cells labeled in flow were captured by the magnet. Laser irradiation of the area near the magnet generates PA signals, which were detected with an ultrasound transducer attached to the tube. Simultaneously, laser irradiation at higher energy allowed PT killing of targeted bacteria. Conventional transmission imaging made it possible to simultaneously control the positions of the laser beam, magnet, and transducer. High-speed imaging also allowed visualization of individual moving cells at the single-cell level as shown in the images (right, bottom) obtained at different magnifications (4×, 20×, and 100×, respectively). Thus, the extracorporeal (bypass) schematic could provide continuous PA monitoring of blood flow in external tubes, and permit efficient capture of abnormal objects (e.g., bacteria or its toxins targeted by MNPs directly in the extracorporeal flow). Magnetic capture of both abnormal objects and unbound magnetic nanoparticles prevents them from being transported further in the systemic circulation. (TIF) [file pone.0045557.s012.tif]
